# Highly Flexible, Self-Bonding, Self-Healing, and Conductive Soft Pressure Sensors Based on Dicarboxylic Cellulose Nanofiber Hydrogels

**DOI:** 10.1021/acsapm.3c01024

**Published:** 2023-08-08

**Authors:** Ragab Abouzeid, Mohammad Shayan, Tongyao Wu, Jaegyoung Gwon, Timo A Kärki, Qinglin Wu

**Affiliations:** † School of Renewable Natural Resources, Louisiana State University, AgCenter, Baton Rouge, Louisiana 70803, United States; ‡ Cellulose and Paper Department, National Research Centre, 33 Bohouth St., Dokki, Giza 12622, Egypt; § Department of Electrical and Computer Engineering, Louisiana State University, Baton Rouge, Louisiana 70803, United States; ∥ Forest Products Department, National Institute of Forest Science, 57 Hoegiro, Dongdaemun-gu, Seoul 02455, Korea; ⊥ Mechanical Engineering Department, Lappeenranta−Lahti University of Technology, Lappeenranta53850 ,Finland

**Keywords:** dicarboxylic cellulose nanofibers, graphene, polyacrylamide, self-healing, conductive hydrogel

## Abstract

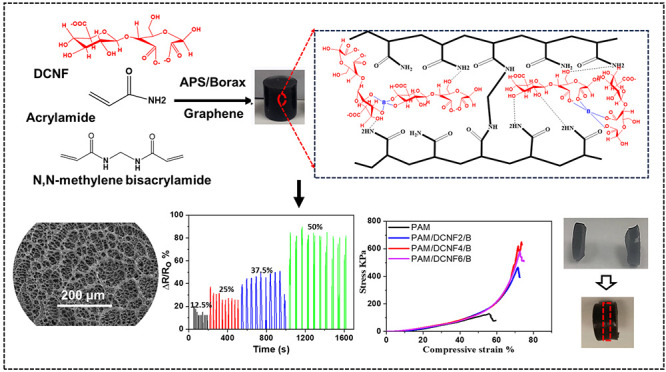

Conductive hydrogels have gained a great deal of interest in the flexible electronics industry because of their remarkable inherent properties. However, a significant challenge remains for balancing hydrogel’s conductivity, self-healing, and strength properties. Herein, double network ionic hydrogels were fabricated by concurrently introducing borax into dicarboxylic cellulose nanofiber (DCNFs) and polyacrylamide (PAM) hydrogels. The incorporation of borax provided a superabsorbent feature to the PAM/DCNF hydrogels (without borax) with the equilibrium water absorption rate increased from 552 to 1800% after 42 h. The compressive strength of the prepared hydrogel was 935 kPa compared to 132 kPa for the PAM hydrogel, with high cycling stability (stable after 1000 compression cycles with 50% strain). The hydrogel pressure sensor had a very sensitive response (gauge factor = 1.36) in the strain range from 10 to 80%, which made it possible to detect mechanical motion accurately and reliably. The developed hydrogels with high-performance, environmentally friendly properties are promising for use in future artificial skin and human–machine interface applications.

## 1 Introduction

Flexible pressure sensors are becoming more popular with the development of science and technology. Sensors for pressure distribution typically use arrays of wires for signal transmission, which limit their usability and flexibility.^1−3^ Nowadays, hydrogel pressure distribution sensors have been developed to overcome these limitations. A hydrogel is a form of a 3D polymer network that can quickly swell in water. Hydrogels contain solid-like polymer networks, while their aqueous phase facilitates rapid carrier diffusion, showing liquid-like transport properties.^4−6^ Due to their biocompatibility and softness, many hydrogels are ideal candidates for applications, including biocompatible materials, drug delivery, energy devices, wound dressings, and tissue engineering.^7,8^ In recent years, hydrogels have been used as sensors in wearable electronics and intelligent systems such as electronic skin, motion monitoring, and physiological monitoring systems. Hydrogels have high stretch and resilience like elastomers and exhibit high biocompatibility and modulus comparable to biological tissues.^9^ Stretchable hydrogel electrodes have been identified as ideally suited for energy storage applications. This is due to their porous structure, tunable chemistry, high electric/ionic conductivity, high specific capacitances, and mechanical robustness. However, the limited compressibility of the flexible hydrogel film severely constrains its application in high-pressure underwater environments. In response, worldwide researchers are working on the development of competent and sustainable energy storage technologies, such as batteries and supercapacitors.^10−13^ Regarding multiscale structures, strengths, and functions, hydrogels demonstrate massive potential for design and processing.^7,14^ In recent years, nanoparticles and double networks have been studied to improve the mechanical strength and strain of the hydrogel.^5,15^ For instance, silica nanoparticle-modified polyacrylamide (PAM) hydrogels have a tensile strength of 70 kPa at 80% strain.^16^ Similarly, chondroitin sulfate-modified PAM hydrogels have a tensile strength of 150 kPa at 80% strain.^17^ Additionally, SC-PDA/GO-Ca^2+^/PAM hydrogels have a tensile strength of 118 kPa at 80% strain,^18^ and polydopamine-coated talc nanoflake-modified PAM hydrogels have tensile strengths of approximately 80 kPa at 70% strain and 300 kPa at 80% strain.^19^ The hydrogels become increasingly resistant to ion migration when their mechanical qualities are increased, making it challenging to maintain high mechanical strength and high conductivity. Despite high conductivity and excellent durability, ionic conductive gels have poor mechanical strength and elasticity.^20,21^ Thus, soft artificial materials must be as strong, elongated, and elastic as human tissue and conductive. Conductive hydrogels, with their water-rich conductive polymer material and 3D network microstructure, can convert mechanical deformation into electrical signals.^22^ Other concerns remain to be addressed, including poor self-healing, skin-adhesive bandages, and low sensitivity. As a result, exploiting a flexible wearable sensor with high sensitivity, self-adhesiveness, and self-healing remains a significant challenge.^23^ Hydrogels that are capable of healing damaged structures and properties have been developed. It has been shown that the development of self-healing hydrogels is mainly driven by noncovalent bonds such as electrostatic interactions, metal–ligand coordination, hydrogen bonds,^24−29^ and invertible dynamic-covalent bonds, including acyl hydrazone bonds, disulfide bonds, imines, and boronic ester bonds.^30−32^

Cellulose nanofibers (CNFs) have excellent mechanical, elastic, and hydrophilicity properties. Oxidization of CNFs introduces a wide range of functionalized groups on the CNFs surfaces. These functional groups enable CNFs to support amino acids, organic macromolecules, and other functionalized organic moieties.^33−35^ Furthermore, these functional groups (e.g., hydroxyl and carboxyl) enhance electrostatic interactions between CNFs and borax, improving the hydrogel’s self-healing properties. Hydrogels based on CNFs have excellent mechanical properties and a highly porous structure. The materials can be readily combined with conductive carbon nanofillers like graphene and carbon nanotubes (CNTs) to prepare wearable motion sensors, leading to thin, flexible, self-healing, and high-performance hydrogels.^36^ The chemical cross-linker borax (sodium tetraborate, Na_2_B_4_O_7_·10H_2_O) is known for its low toxicity, low cost, and water-soluble nature and is an exciting candidate. When borax is dissolved in an aqueous solution, it dissociates into trigonal boric acid (B(OH)_3_) and tetrahydroxy borate ion (B(OH)_4_^–^), both of which react with polymer functional groups, forming dynamic borate-diol bonds.

Developing a conductive flexible hydrogel sensor with high adhesion and sensing stability has proven challenging. This study aims to use dicarboxylic CNFs (DCNFs) with borax, graphene nanoplatelets, and acrylamide to create double network hydrogels with excellent mechanical performance, good shape stability, and self-healing ability. Hydrogels prepared from polyacrylamide (PAM) and the CNF-borax (PAM/DCNF/B) system had a higher self-healing potential than PAM/DCNF hydrogels because of dynamic borate-diol bonds formed. Also, as a result of physical entanglements and hydrogen-bonded interactions, the PAM hydrogel displayed significantly enhanced mechanical properties. The PAM/DCNF and PAM/DCNF/B hydrogels were assembled into a pressure-type strain sensor to detect human motion, demonstrating high sensitivity, linearity, and stable signal feedback.

## 2 Experimental Section

### 2.1 Materials

Bleached bagasse fibers extracted from bagasse were supplied by Qena Pulp and Paper Industry (Qena, Egypt). The bleached bagasse pulp was composed of 70.6% α-cellulose, 29.7% pentosans, and 0.82% ash while exhibiting a degree of polymerization (DP) of 1135.^37^ Sodium periodate (NaIO_4_), sodium hydroxide (NaOH), sodium chlorite (NaClO_2_), graphene nanoplatelets (GNP), acrylamide (AM), ammonium persulfate (APS), and *N*,*N*-methylene bis(acrylamide) (MBA) of laboratory grade were purchased from Fisher Scientific Co. (Hampton, NH, USA). Sodium tetraborate decahydrate (borax) was purchased from Sigma-Aldrich Inc. (St. Louis, MO, USA). All reagents used in this work were of analytical quality, and deionized water was used to prepare the aqueous solutions.

### 2.2 Preparation of DCNFs

Preparation of DCNFs followed a published method.^38,39^ First, 5 g of bleached bagasse pulp was stirred with 4.5 g of NaIO_4_ in 500 mL of water for 24 h in the dark at room temperature. Then, the excess periodate was decomposed using ethylene glycol. The resulting product was washed with deionized water and centrifuged (1500*g*, 5 min), and the process was repeated several times. Dialdehyde cellulose was dried at 50 °C for 12 h, yielding around 90% solid. The oxidation of dialdehyde cellulose with sodium chlorite (1 M) in acetic acid solution at pH = 4 for 24 h was carried out. Afterward, the oxidized product was washed with deionized water several times and stored at 4 °C in a freezer. A consistency of 0.5% of oxidized cellulose was dispersed in deionized water, and the pH of the suspension was adjusted to ∼7.5 using NaOH. A high-pressure homogenizer was used to produce dicarboxylic cellulose nanofiber suspensions (model M-110EH-30 microfluidics; Microfluidics Corp., Newton, MA, USA).

Conductimetric titration was used to determine the carboxyl content of DCNFs.^39,40^ Briefly, dry DCNFs samples (50 mg) were suspended in 0.01 M HCl for 2 h to exchange Na^+^ ions with H^+^ protons. Conductivity was measured every 0.5 mL of NaOH solution titrated with 0.01 M NaOH solution. The carboxylate content of DCNFs was measured from the sudden change in conductivity. Equation 1 was used to calculate carboxylate content (mmol/g):
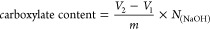
1where *V*_1_ and *V*_2_ correspond to the volumes of NaOH solution (mL) used from neutralizing the added HCl and carboxylic acid on the DCNFs. *N*_(NaOH)_ corresponds to the NaOH solution concentration (M), and *m* is the weight of the dried sample (g).

### 2.3 Synthesis of PAM/DCNF and PAM/DCNF/B Hydrogels

PAM/DCNF hydrogels were synthesized via free radical polymerization of AM in an aqueous suspension containing DCNFs and graphene nanoplatelets (GNP) using MBA as a cross-linker and APS as an initiator. A typical procedure for dispersing GNP with DCNFs in water was to mix and stir the mixture for 15 min, followed by sonication for another 15 min. The AM monomer and MBA cross-linkers were added to the homogeneous suspension of DCNF/GNP with continuous stirring for 1 h. After that, APS was added, and the mixture was stirred for 15 min. The mixture suspension was transferred into a circular mold after being sonicated for 1 h to remove air bubbles. The suspensions were placed in an oven at 40 °C for 6 h to induce polymerization. In other experiments, the same procedures were performed with the addition of borax solution. The formulations of all samples prepared are listed in Table S1.

### 2.4 Characterization

A benchtop freeze-dryer (FreeZone 4.5 L -50C, Labconco, Kansas City, MO, USA) was used to lyophilize the hydrogels at −50 °C for 4 days. Infrared spectroscopy (FTIR) analysis of the dried gels in the 4000–500 cm^–1^ domain was conducted using a Tensor 27 FTIR analyzer (Bruker Corporation, Billerica, MA, USA). This analysis had a 4 cm^–1^ scanning resolution and was done in ATR mode. Raman measurements were performed at room temperature using Raman microscopes (Renishaw, UK) under the excitation wavelength of 532 nm. To investigate the crystalline nature of the hydrogels with and without borax, X-ray diffraction (XRD) analysis was performed using a PANalytical Empyrean X-ray Diffractometer (Malvern Panalytical Ltd., Malvern, Worcestershire, UK) equipped with a CuKα radiation source (λ = 1.5406 Å). The operated scattering angle range was from 4° to 80°. A rheometer (AR-2000ex, TA Instruments, New Castle, DE, USA) was used to characterize the rheological properties of the samples. A disk of 25 mm in diameter and 4 mm in thickness was cut out of hydrogel. Dynamic frequency sweeps were performed between 0.1 and 100 rad/s at 0.1% strain amplitude. After that, the strain range was changed from 0.01 to 100% and the amplitude sweep was performed at a constant frequency of 10 rad/s. The morphology of hydrogels was analyzed by a field emission-scanning electron microscopy (FE-SEM) Quanta 3D DualBeam FEG FIB-SEM (FEI Co., Eindhoven, Netherlands) using 5 kV acceleration voltage. The DCNFs prepared were characterized with a JEM 1400 Transmission Electron Microscope (Peabody, MA, USA).

Thermogravimetric analysis (TGA) of hydrogels prepared with and without borax was conducted using a Q50 Analyzer (TA Instruments Inc., New Castle, DE) in a nitrogen atmosphere. The temperatures ranged from 30 to 600 °C, with heating rates of 10 °C/min, and the sample weight was 5 mg.

Mechanical properties of the hydrogels were measured with an Instron 5900R testing machine (Instron Corp, Norwood, MA, USA) at a compression speed of 10 mm min^–1^ at room temperature. The hydrogel samples (25 mm in diameter and 20 mm in height) were subjected to compressive stress with a 90.7 kg loaded cell. Piezoresistive sensing performance was measured by using an eight-channel LAND battery analyzer (CT3001A, LAND Electronics Corporation, Wuhan, China). Two conductive metals were attached to both sides of the hydrogels to analyzers to obtain the output of the electrical signals. Equation 2 was used to calculate the relative change of resistance based on measured current. The gauge factor (GF) was calculated using eq 3.^41^
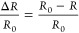
2
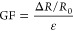
3where *R*_0_ and *R* represent the resistance of the sample before and after pressing, respectively, and ε is the applied strain.

The impedance measurements were conducted on an electrochemical workstation (CHI650E B14109, Chen Hua, China). Hydrogel samples with a diameter of 25 mm and a length of 4 mm were clamped and attached to electrodes on the electrochemical workstation. A.C. impedance testing was performed at a frequency of 0.01–10000 Hz with a disturbance voltage of 10 mV. Hydrogels were also attached to human fingers and then connected to a digital display sensing test system (LAND battery analyzer; CT3001A, LAND Electronics Corporation, Wuhan, China) using copper tape and wires. Current changes were measured to test the effectiveness of human motion monitoring.

### 2.5 Water Absorption Behaviors

Water absorption (WA) was determined gravimetrically. Dry hydrogels were soaked in distilled water for a specific period. To remove free water from the sample surface after they were removed from the water, the samples were quickly wiped with tissue paper and then weighed again.

The following equation was used to calculate the water absorption (WA, %):
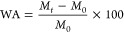
4where *M*_0_ is the initial mass of the dry hydrogels (g), and *M_t_* is the mass of the hydrogels (g) at swelling time *t*.

The following equation was used to calculate fractional water absorption (FWA):
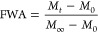
5where *M*_0_ is the initial weight of the dry hydrogels (g), *M_t_* is the weight of the hydrogels at time *t* (g), and *M*_∞_ is the equilibrium weight of the hydrogels (g). FWA data were plotted against the square root of time to show different trends.

## 3 Results and Discussion

### 3.1 Fabrication and Basic Properties

**Figure 1:**
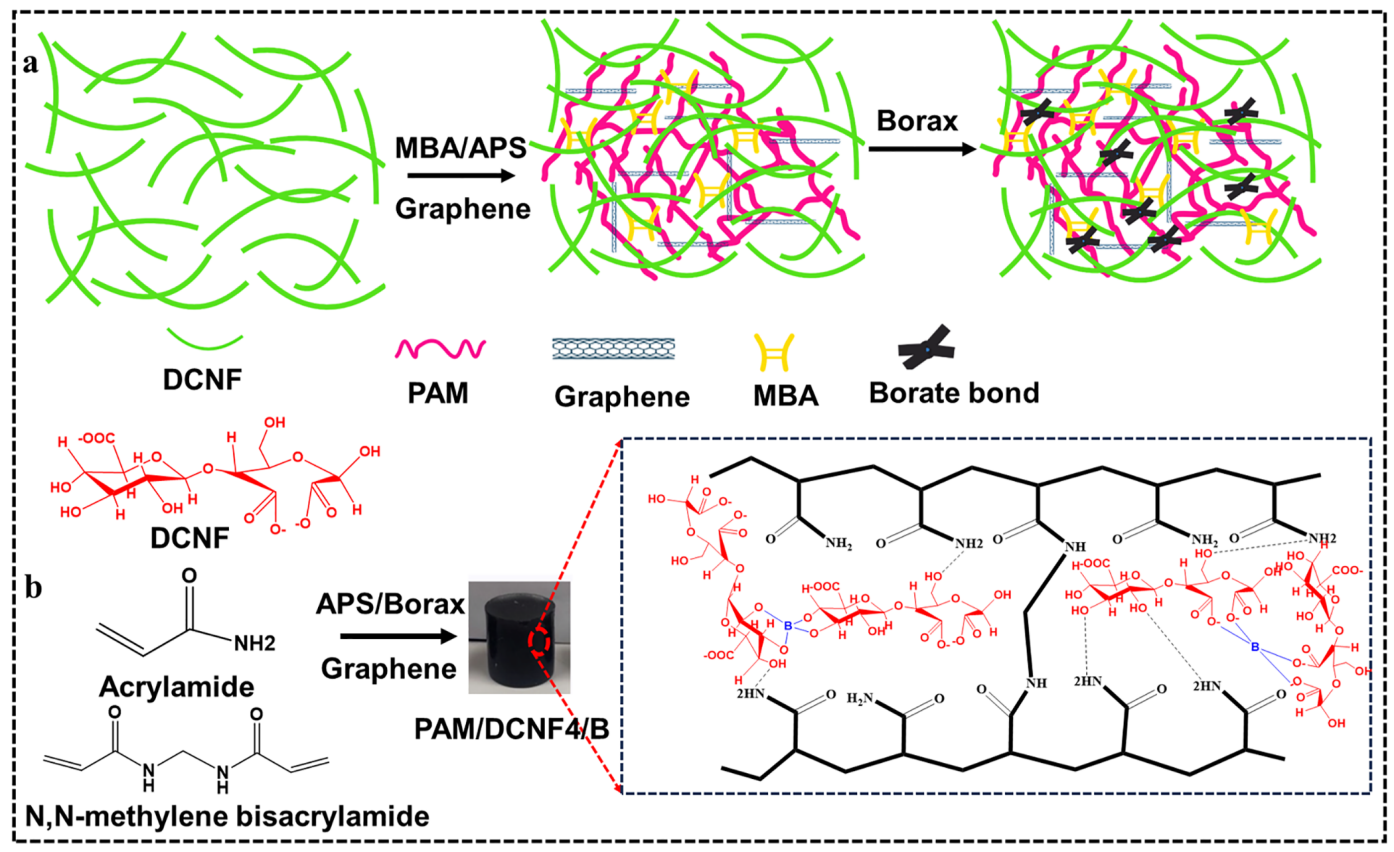
Schematic diagram of (a) preparation of hydrogels using dicarboxylic cellulose nanofibers (DCNFs), graphene nanoplatelets (GNP), acrylamide (AM), ammonium persulfate (APS), and *N*,*N*-methylene bis(acrylamide) (MBA) and (b) suggested mechanism of the hydrogel formation, illustrating the interaction of DCNFs, GNP, AM, MBA, and APS.

Figure 1 demonstrates the procedure for the preparation of the hydrogel. A stable colloidal suspension was first formed by dispersing DCNFs in an aqueous suspension containing graphene nanoplatelets with or without borax. Then, the dispersion was stirred with acrylamide (AM), initiator (APS), and cross-linker (MBA) for 2 h. The hydrogel is formed by free radical polymerization of acrylamide, with *N*,*N*′-methylene bis(acrylamide) as a cross-linker and ammonium persulfate as an initiator (Figure 1b). Finally, the homogeneous mixture suspensions were placed in a glass tube and inserted into an oven at 60 °C to create PAM/DCNF and PAM/DCNF/B hydrogels. The FTIR spectra of cellulose pulp and DCNFs are shown in Figure S1. After oxidation, the appearance of a new peak at 1750 cm^–1^ corresponds to the C=O groups due to oxidation.

Figure S2a,b shows the transmission electron microscopy (TEM) image and conductometric titration of the prepared DCNFs, respectively. The TEM image reveals that the diameter of the individual nanofibers is estimated to be between 10 and 20 nm. The titration curves observed in the experiment exhibited a parabolic shape relationship with the amount of NaOH added. This indicated that the initial decrease in conductivity was due to the neutralization of HCl with NaOH and was not related to the charge nature of DCNFs. Once the neutralization was complete, the conductivity plateaued and the NaOH consumption was associated with the weak carboxylic acid present on the oxidized DCNFs. The charges associated with the carbonyls were calculated from the NaOH consumed in the plateau regions to be 2.34 mmol/g of cellulose.

**Figure 2:**
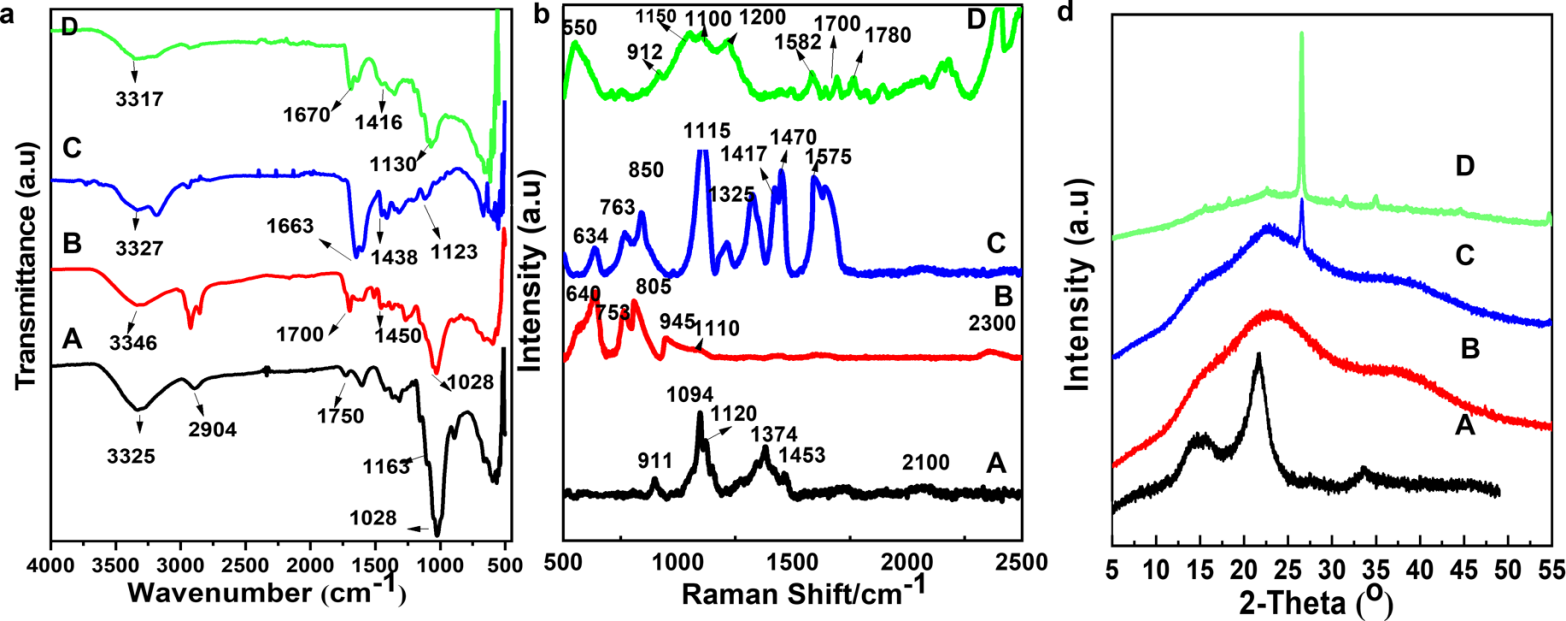
FTIR (a), Raman spectra (b), and XRD (c) spectra of (A) DCNFs, (B) PAM, (C) PAM/DCNF4, and (D) PAM/DCNF/B hydrogels.

**Figure 3:**
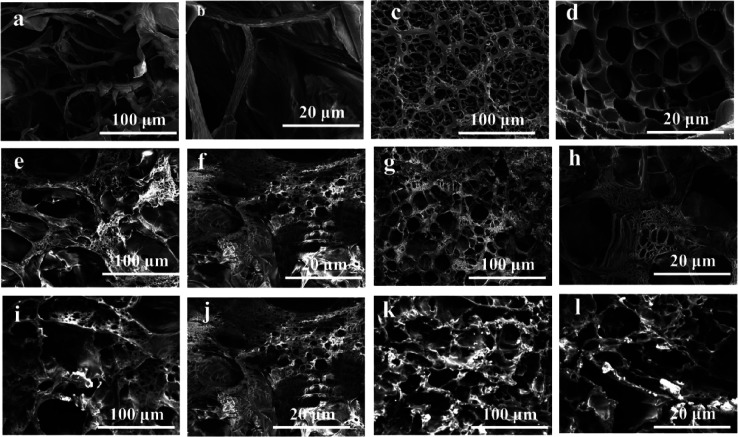
SEM images of the cross section of PAM hydrogels and different concentrations of DCNFs without borax at 100 and 20 μm magnifications. (a, b) DCNFs, (c, d) PAM, (e, f) PAM/DCNF2 (2 wt % DCNFs), (g, h) PAM/DCNF4 (4 wt % DCNFs), (i, j) PAM/DCNF2/B (2 wt % DCNFs with borax), and (k, l) PAM/DCNF4/B (4 wt % DCNFs with borax) hydrogels.

The FTIR, Raman spectroscopy, and XRD of DCNFs, PAM/DCNF, and PAM/DCNF/B hydrogels are presented in Figure 2a–c, respectively. Figure 2a displays the FTIR spectra of DCNFs, PAM, PAM/DCNF4, and PAM/DCNF4/B. The absorption peaks observed in the FTIR spectra of DCNFs at 3325, 1163, and 1028 cm^–1^ correspond to the molecular vibrations of OH stretching, C–O–C stretching, and C–O stretching, respectively, which are characteristic of cellulose.^13,42^ The absorption peak at 1750 cm^–1^ exhibits the stretching vibration of the C=O group, implying the successful oxidation of cellulose.^12^ On the other hand, the PAM spectrum shows a distinct peak at 3346 cm^–1^, which corresponds to the N–H asymmetric stretching. The peaks at 1700 and 1450 cm^–1^ correspond to the C=O stretching of the amidic group and the C–N stretching, respectively. Due to the overlap between DCNFs characteristic absorption peak and PAM-related absorption peak, the PAM/DCNF4 and PAM/DCNF4/B hydrogel peaks do not change significantly in comparison with these for pure PAM hydrogel. The broad absorption band of N–H stretching moving from 3325 to 3304 cm^–1^ is due to hydrogen bonds formed between DCNFs and PAM, and this moving also results from overlapping of the O–H stretching of DCNFs and N–H stretching. The peak value of C=O stretching vibration of PAM/DCNF hydrogels varies from 1750 to 1650 cm^–1^ due to intermolecular and/or intramolecular interactions between PAM and DCNFs, such as hydrogen bonding or van der Waals forces.^43^ Borate ions can cross-link the polymer chains in the hydrogel, which can lead to changes in the vibrational modes of the chemical functional groups. The absence of peaks at 1059 cm^–1^ in the PAM/DCNF4 and PAM/DCNF4/B hydrogel spectra indicates the interaction between PAM and DCNFs. These interactions and cross-linking were further supported by the observed changes in the SEM images (Figure 3), such as the reduction in pore size and porosity, which indicated the formation of a more compact structure within the hydrogel network.

To further confirm the structure of the hydrogels, the Raman spectra were conducted for DCNFs, PAM, PAM/DCNF4, and PAM/DCNF4/B. The spectrum of DCNFs indicated C–H and C–H_2_ stretching as well as asymmetric stretching of the C–O–C glycosidic linkage at 1094 cm^–1^. Additionally, peaks of H–C–H bending and H–O–C bending were observed at 1453 and 1374 cm^–1^, respectively. In the case of the PAM hydrogel, the amide band at 1110 cm^–1^ was a combination of N–H bending, C–N stretching, and C–C stretching vibrations, while the peak at 950 cm^–1^ was likely due to C–C stretching vibrations in the polyacrylamide backbone, and the peak at 805 cm^–1^ was attributed to N–H bending vibrations in the amide groups. For PAM/DCNF4, which contains 4% DCNFs and 1% graphene, the G band at 1550 cm^–1^ was present, corresponding to the in-plane vibration of sp^2^ carbon atoms in graphene as well as the D band at 1335 cm^–1^, indicating defects or disorder in graphene. Finally, in the presence of borax in PAM/DCNF4/B, the bending vibration peaks of −O–B–O– at 550 cm^–1^ and of BO–H at 1100 cm^–1^ were observed. The X-ray diffraction patterns of DCNFs, PAM, PAM/DCNF4, and PAM/DCNF4/B are displayed in Figure 2b. The hydrogels showed an amorphous morphology based on their spectra. Diffractograms of DCNFs exhibited characteristic cellulose I peaks at 2θ = 14°, 16°, 22.5°, and 34°, corresponding to crystallographic planes (101), (10̅), (200), and (040). PAM shows an amorphous structure in its diffraction diffractogram. In PAM/DCNF4 and PAM/DCNF4/B hydrogels, the peak at 2θ = 26.5° corresponding to crystallographic plane 002 is for graphene nanoplatelets. The hydrogel containing DCNF with PAM and graphene demonstrated a decrease in the intensity of the peaks assigned to crystalline cellulose and an increase in the broad peak, indicating amorphous cellulose.

The thermal stability of PAM-based hydrogels with and without borax was studied with TGA curves shown in Figure S3 for all hydrogels.

### 3.2 Morphological Properties

The internal network structures were characterized by SEM of DCNFs, PAM, PAM/DCNF2, PAM/DCNF4, PAM/DCNF2/B, and PAM/DCNF4/B hydrogels with two different magnifications, as shown in Figure 3. Figure 3a,b displays the SEM pictures of dry DCNFs, which reveal a dense network structure, whereas Figure 3c,d displays the SEM images of PAM, indicating a more porous structure. It is shown that the hydrogels exhibit an excellent continuity of micropore structure, and their surfaces are flexible and smooth, forming a solid network structure to absorb water, which is consistent with the results from previous work.^9,17^ The pore size and porosity decreased compared to the PAM hydrogels with the addition of different concentrations of DCNFs (Figure 3e–l). ImageJ software was used to determine the pore size of PAM, PAM/DCNF2, and PAM/DCNF2/B, which are 12.1 m, 9.12 μm, and 8.23 μm, respectively. As a result of the interaction between the DCNFs and the PAM chains, a more compact structure was obtained.^44^ This can be attributed to the hydrogen-bonding interaction between DCNF-containing carboxyl and hydroxyl groups and the polar functional groups of the PAM side chains. When DCNFs are added to PAM hydrogels, they can interact with the PAM chains through various mechanisms such as hydrogen bonding, electrostatic interactions, or van der Waals forces. These interactions can lead to a more compact structure in the hydrogel, which can result in a decrease in pore size and porosity. The addition of borax to the chemical composition of the hydrogel increases the interactions among the components, leading to a denser and more compact structure. This denser structure is beneficial for hydrogels used in applications such as soft pressure sensors for human motion, as it increases the hydrogel’s mechanical stability. The correlation between the FTIR and SEM analyses reveals how the chemical composition affects the structural features of the hydrogels such as the formation of hydrogen bonds, the pore size, and the porosity.

### 3.3 Rheological Properties

**Figure 4:**
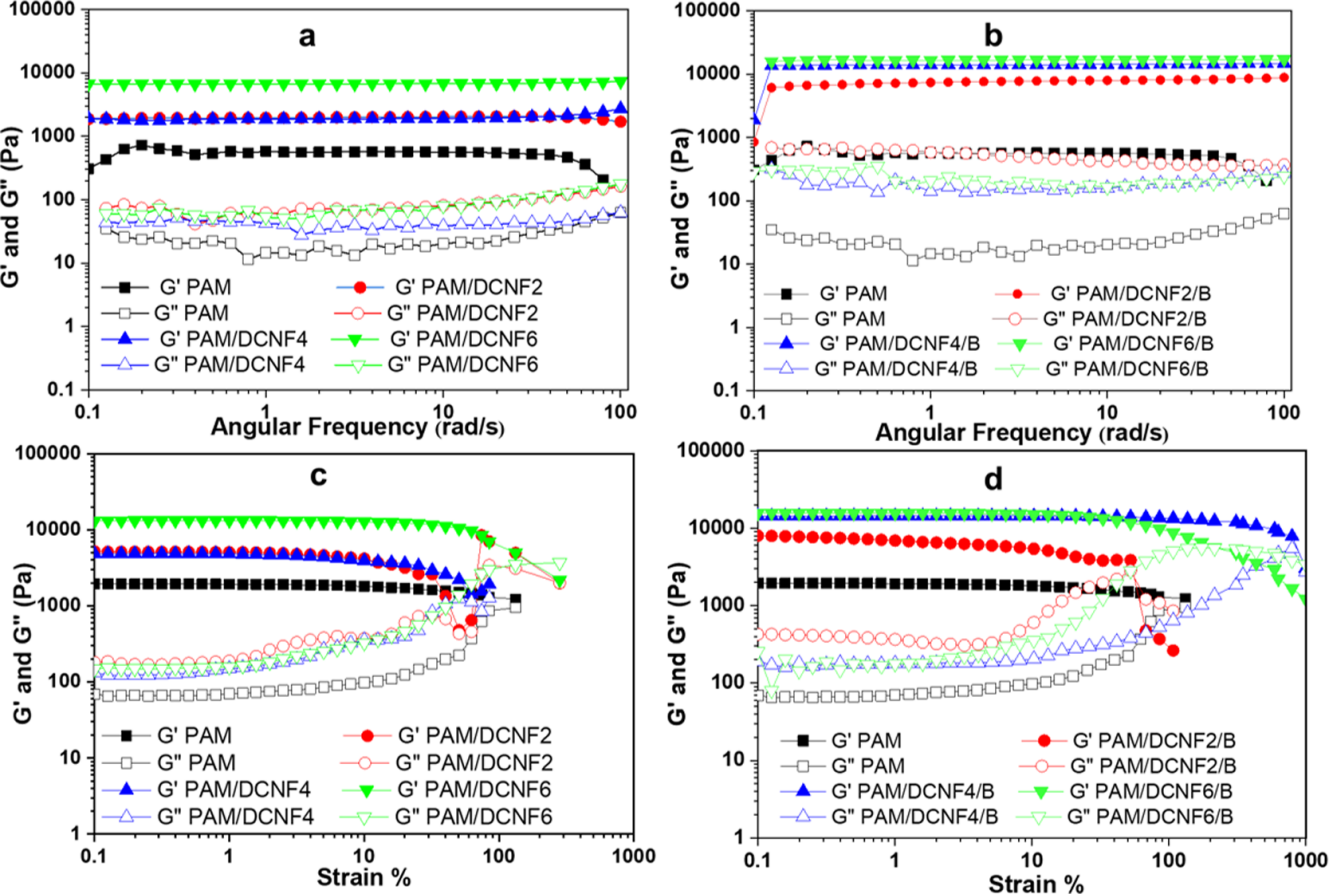
Effect of DCNF content on the dynamic viscoelasticity performance of the hydrogels at 25 °C. (a, b) Dynamic frequency curves at 0.1% strain amplitude. (c, d) Dynamic strain curves at a constant frequency of 10 rad/s.

The viscoelastic properties of hydrogels were evaluated in a frequency sweep ranging from 0.1 to 100 rad/s at a constant strain of 0.1% and 25 °C for the hydrogels with and without borax (Figure 4a,b). The PAM, PAM/DCNF2, PAM/DCNF4, and PAM/DCNF6 hydrogels showed no noticeable change in storage modulus (*G*′) and loss modulus (*G*″) when the frequency changed from 0.1 to 100 rad/s. In addition, the *G*′ of the hydrogels was always larger than the *G″*, confirming that the hydrogels had formed a cross-linked network. However, for the PAM/DCNF/B hydrogels, within the whole angular frequency range, *G*′ and *G*″** curves were higher than those of PAM/DCNF hydrogels. Furthermore, *G*′ was greater than *G*″** during the oscillatory process, demonstrating a rather stable and strong double network of the PAM/DCNF/B hydrogels. Figure 4c,d shows the strain sweep at a constant frequency of 10 rad/s. It was found that the PAM/DCNF hydrogels maintained their cross-linked network until the strain was less than 100%. At the same time, the PAM/DCNF/B hydrogels continued their cross-linked network until the strain was larger than 590%, especially for PAM/DCNF4/B and PAM/DCNF6/B. This is because the density of the cross-linked network was higher in the hydrogels with borax than those without borax.

The graphs of complex modulus (G*) and complex viscosity (η*) versus frequency provided an apparent contrast between PAM/DCNF and PAM/DCNF/B hydrogels (Figure S4a,b). The G* and η* of all hydrogels almost completely overlapped, meaning that the hydrogels acted like a viscous fluid. Meanwhile, the G* and η* plots, as well as the data in Figure S5a,b, show the loss tangents (tan δ) of PAM/DCNF and PAM/DCNF/B hydrogels. The loss tangents of PAM/DCNF/B are all much higher than those of PAM/DCNF, indicating a robust network structure of PAM/DCNF/B hydrogels due to the presence of borax.

### 3.4 Water Absorption Properties

**Figure 5:**
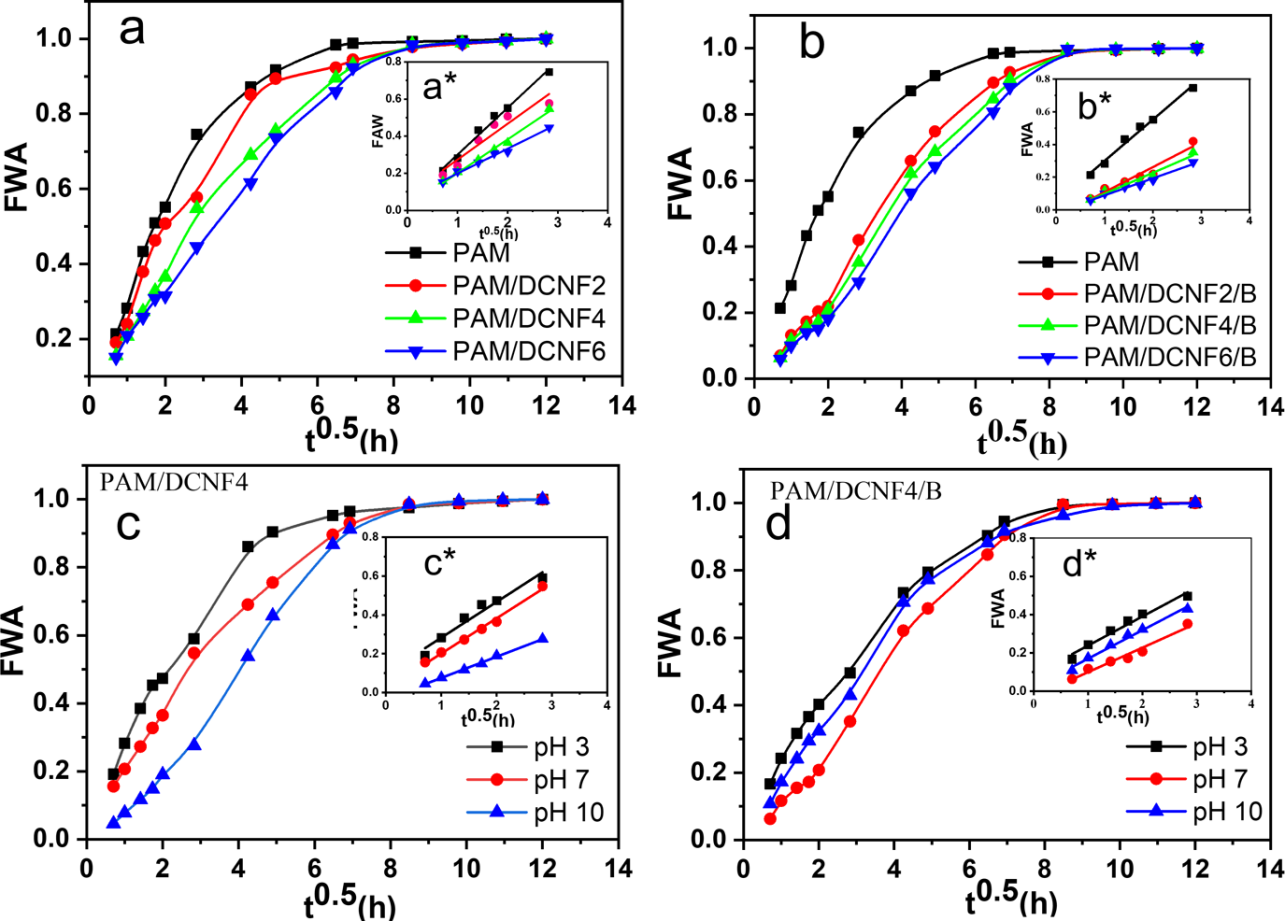
Fractional water absorption as a function of square root of time for PAM/DCNF (a) and PAM/DCNF/B hydrogels (b) and effect of different pH levels of PAM/DCNF4 (c) and PAM/DCNF4/B hydrogels (d). Linear FWA in the initial stages of absorption for all hydrogels (a*, b*, c*, and d*).

Conductive hydrogels are hydrophilic materials that can absorb and retain large amounts of water. Their WA properties can significantly affect their applications such as human motion sensors, in particular, the sensor’s sensitivity, response time, mechanical strength, and stability. Figure 5 shows FWA plotted against the square root of soaking time (h^1/2^) for various hydrogels at three different pH levels. The WA rate, represented by the initial slope of the FWA curve, was affected by the hydrogel composition and pH. The FWA values showed a linear relationship in the early stages of absorption as shown in Figure 5a*–d*. For PAM and PAM/DCNF6, the slope decreased from 0.25 to 0.13, indicating that the increase in DCNF content in hydrogels led to reduced WA rate due to higher cross-linking density and lower porosity in the hydrogel. In contrast, in the presence of borax, the cross-linking density of the hydrogel increased, and the pore size within the hydrogel decreased, making it more difficult for water to enter and penetrate the hydrogel network. This resulted in a lower initial WA rate (i.e., reduced initial slope of the FWA curve from 0.25 to 0.10 for PAM and PAM/DCNF6/B). However, once the hydrogel network was penetrated by water, its ability to hold water within its structure was increased by the additional cross-linking provided by borax. This resulted in an enhanced water absorption capacity. Additionally, the reaction between borax and DCNFs induced an increased number of functional hydroxyl groups, such as the monodical-borax complex, which interacted with water, further contributing to the increased water absorption capacity.^45^ The FAW slope was observed to decrease at the beginning of the test for PAM/DCNF4 from 0.184 to 0.11 for pH from 3 to 10, indicating a lower rate of water absorption. After that, the water absorption increased dramatically with increasing pH due to the carboxylate groups of the hydrogel being ionized to COO^–^ ions, and the hydrogel network chain repulsion increased, causing higher water uptake. At lower pH levels, protonation of carboxylate groups of the hydrogel led to a decrease in the main anion–anion repulsive forces and resulted in a decrease in WA.^38,46−49^ This phenomenon was described in a recent study of carboxymethyl cellulose-*g*-poly(acrylic acid-*co*-acrylamide) superabsorbent hydrogels.^50^ After approximately 40 h, all hydrogel samples achieved a state of saturation, as evidenced by the FWA values no longer increasing over time, attributed to more physical cross-linking and less porosity.

### 3.5 Mechanical Performance

**Figure 6:**
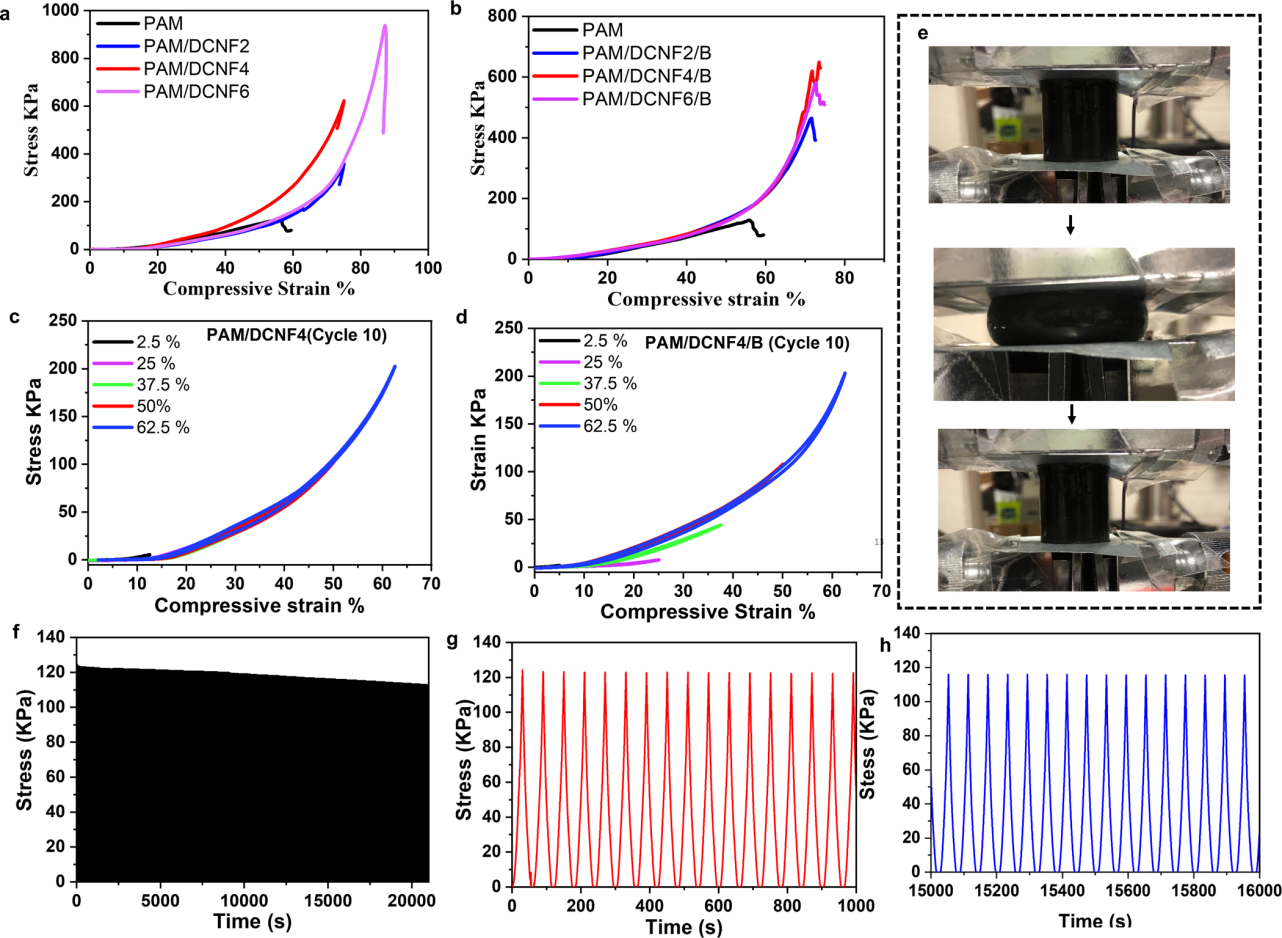
Compressive strength properties of the hydrogel samples: (a, b) compressive stress–strain curves for PAM/DCNF and PAM/DCNF/B hydrogels, respectively; (c, d) cycle 10 of continuous cyclic compressive stress–strain curves of PAM/DCNF4 and PAM/DCNF4/B hydrogels, respectively; (e) compressing and recovery process of the PAM/DCNF4B hydrogel; (f–h) 1000 successive cycle loading–unloading curves during the compression test for PAM/DCNF4/B.

The strength of the PAM/DCNF and PAM/DCNF/B hydrogels under compression is shown in Figure 6a,b. When compared to PAM hydrogels, the compressive strength of PAM/DCNF hydrogels was seven times stronger, with values of 132, 360, 622, and 935 kPa for PAM, PAM/DCNF2, PAM/DCNF4, and PAM/DCNF6 hydrogels, respectively. This indicates that incorporating DCNFs into PAM hydrogels significantly increased their compression strength. A similar effect was observed with PAM/DCNF/B hydrogels due to the formation of more hydrogen bonds between DCNFs and PAM. Furthermore, Figure 6c,d and Figure S6a,b show that PAM/DCNF4 and PAM/DCNF4/B hydrogels maintain their compressive strength ranging from 24 to 208.5 kPa, even when subjected to strain rates of 2.5 to 80% in the first and tenth cycles. This allows the hydrogels to function within a wide range of pressures. The hydrogel consisting of graphene oxide and *N*,*N*-isopropyl diacrylamide was developed for use in a highly sensitive capacitive pressure sensor. The resulting hydrogel exhibited a breaking strength of 42.20 kPa and a compressive strength of 84.17 kPa after undergoing 50 cycles.^51^ In another study, a self-adhesive hydrogel for wound healing was created by using polyacrylamide (PAM), naturally derived chitosan (CS), and tannic acid/ferric ion chelates. The compressive strength of the hydrogel was found to be approximately 371 kPa at a strain of 80%, which was higher than that of the PAM hydrogels (196 kPa) but lower than that of the PAM/CS hydrogels (516 kPa).^52^ Also, a superior stretchability and toughness hydrogel for the delivery of alendronate was reported, which could have potential applications in tissue engineering. The compressive strength of the hydrogels at the strain of 80% was the highest at 209.47 kPa.^53^ In a comparison with the CNF hydrogel, Valentina et al. investigated the mechanical properties of CNF (cellulose nanofiber) hydrogels using compression tests. This study shows that an increase in CNF solid content led to an increase in compression stress, indicating a higher resistance to deformation and a greater stiffness of the hydrogels.^54^ The cyclic tests, which involve applying repeated cycles of load and unloading, demonstrated that PAM/DCNF4 and PAM/DCNF4/B hydrogels are capable of recovering from deformation. Figure 6f–h illustrates an example of PAM/DCNF4/B hydrogels that can withstand compression strains of up to 50%. The stress–time curves from the cycle loading–unloading tests did not indicate any significant decrease in stress, even after 1000 cycles. The presence of DCNFs in the PAM network structure reduced the formation of microcracks during loading and unloading cycles, while the borate ester bond formed with DCNFs extended the hydrogel’s lifetime and facilitated self-healing. Moreover, the cellulose-based composite exhibited superior strain resistance, as indicated by a recovery rate of over 95%. Figure 6e demonstrates the compression and recovery process of the PAM/DCNF4B hydrogel after being subjected to 80% strain. This suggests that the hydrogels are capable of withstanding large strains without breaking. These findings highlight the superior mechanical properties of PAM/DCNF4/B hydrogels, making them promising candidates for use in wearable and extensible sensors.

### 3.6 Conductivity and Sensing Performance

**Figure 7:**
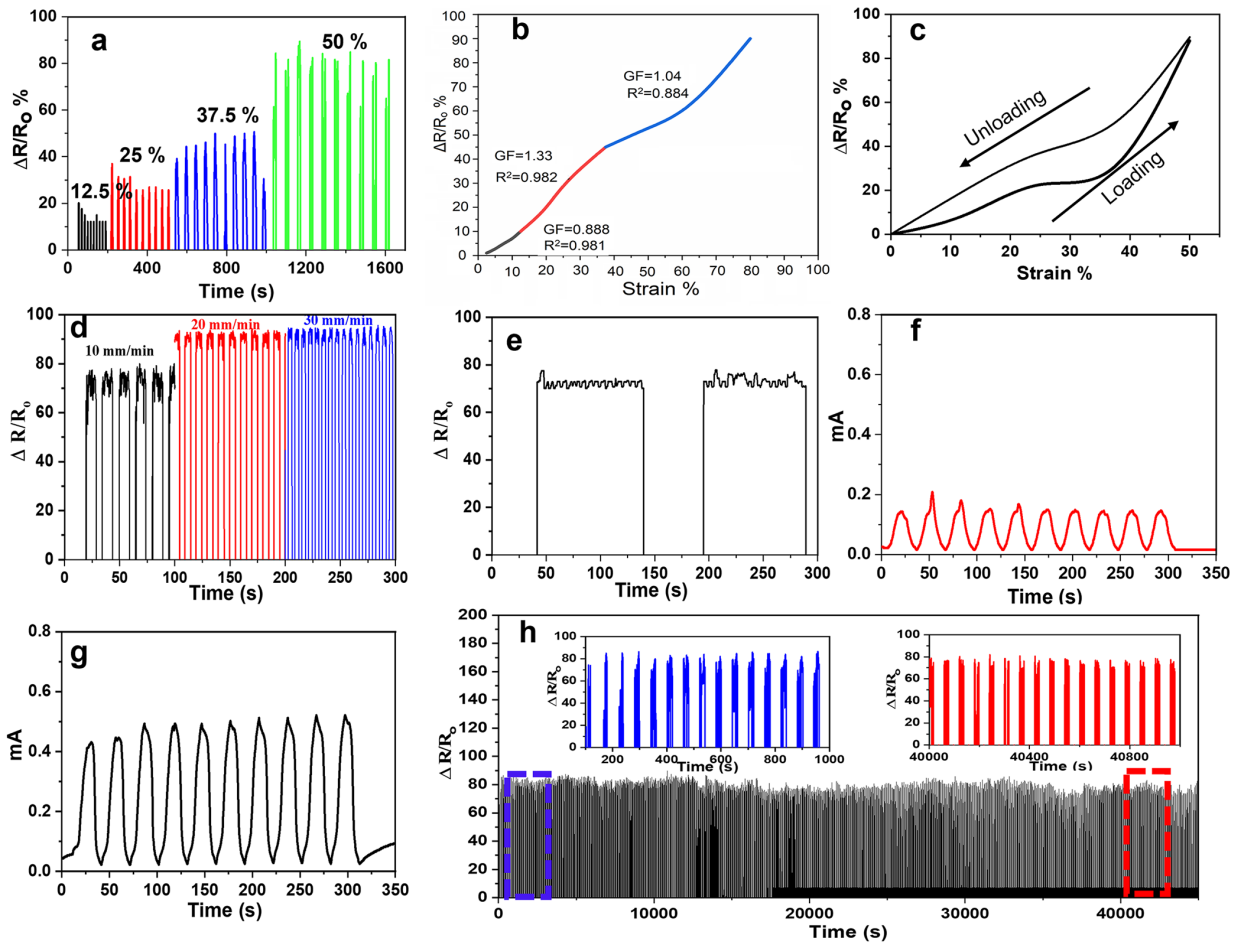
Sensor performance of the PAM/DCNF4/B hydrogel: (a) related real-time sensing property reacting to different strains (12.5, 25, 37.5, and 50%), (b) gauge factor of the PAM/DCNF4/B hydrogel under applied strain, (c) relative resistance change during the loading–unloading cycle at 50% strain rates, (d) relative resistance change curve with different compression test speeds at 50% strain, and (e) response time of the strain sensor with loading and unloading. (f, g) Current intensity change at 25% strain of PAM/DCNF4 and PAM/DCNF4/B, respectively. (h) Relative resistance change curve of the PAM/DCNF4/B hydrogel under 1000 compression cycles at 50% strain.

The PAM/DCNF/B hydrogel could be an excellent pressure sensor owing to its strain-dependent resistance, mechanical compressive strength, and excellent recyclable compressibility. Figure 7a illustrates a real-time sensing performance at different strains. The PAM/DCNF4/B system, as an example, demonstrates a wide range of detection capabilities with promising properties. PAM/DCNF4/B was systematically studied for its piezoresistive performance. With strains ranging from 12.5 to 50%, the sensor provided a clear and distinct response at every strain or pressure level, demonstrating its high sensitivity. There was a linear tendency between the relative resistance change (Δ*R*/*R*_o_) and applied strain. Thus, the gauge factor (GF) was calculated using eq 3 to determine the strain sensor’s sensing ability. The GF data are plotted as a function of strain and resistance change in Figure 7b. The GF values can reach 1.33 at a range of strains comparable to that reported by other strain sensors. First, the GF was 0.88 for strains below 12.5% but increased to 1.33 from 12.5 to 50%. Furthermore, GF decreased to 1.04 for strains between 50 and 80%. Compared with other pressure sensors, the prepared hydrogel sensors exhibit high sensitivity.^55−57^Figure 7c illustrates the loading and unloading curves of the hydrogel at 50% strain rates, highlighting its excellent elastic recovery capabilities. In addition, Figure 7d shows that the relative resistance changes with the changes in the compression speed tests. The output signal is independent of motion frequency, and its shape remains almost uniform when detecting irregular human behavior, which is more important for detecting irregular findings. It is demonstrated that the PAM/DCNF4/B hydrogel has excellent electrical stability as well as outstanding sensitivity, making it suitable for use in flexible wearable electronic devices. It is also shown in Figure 7e that the sensor responded and recovered in real time, which makes it possible to detect strain in real time using such a wearable strain sensor.^58^ Further, the currents of the prepared hydrogels (PAM/DCNF4 and PAM/DCNF4/B) were measured during the compression test with 25% strain (Figure 7f,g). When the current of PAM/DCNF4/B was compared with that of PAM/DCNF4, it was observed that the current increased in the presence of borax due to the increase in electron migration in the hydrogel. The hydrogels’ impedance tests (Figure 9) further confirmed this behavior. To study the ability of the pressure sensors during the long cycles of compression, the relative resistance change was tested with 1000 cycles of loading and unloading with 50% strain. As observed in Figure 7h, the peaks of the current maintained stability during these long-term cycles. After the cyclic strain, no noticeable deterioration of the Δ*R*/*R*_0_ was found. Considering the high electronic sensitivity and long-term durability of the hydrogel used as a pressure sensor, the hydrogel has considerable potential.

**Figure 8:**
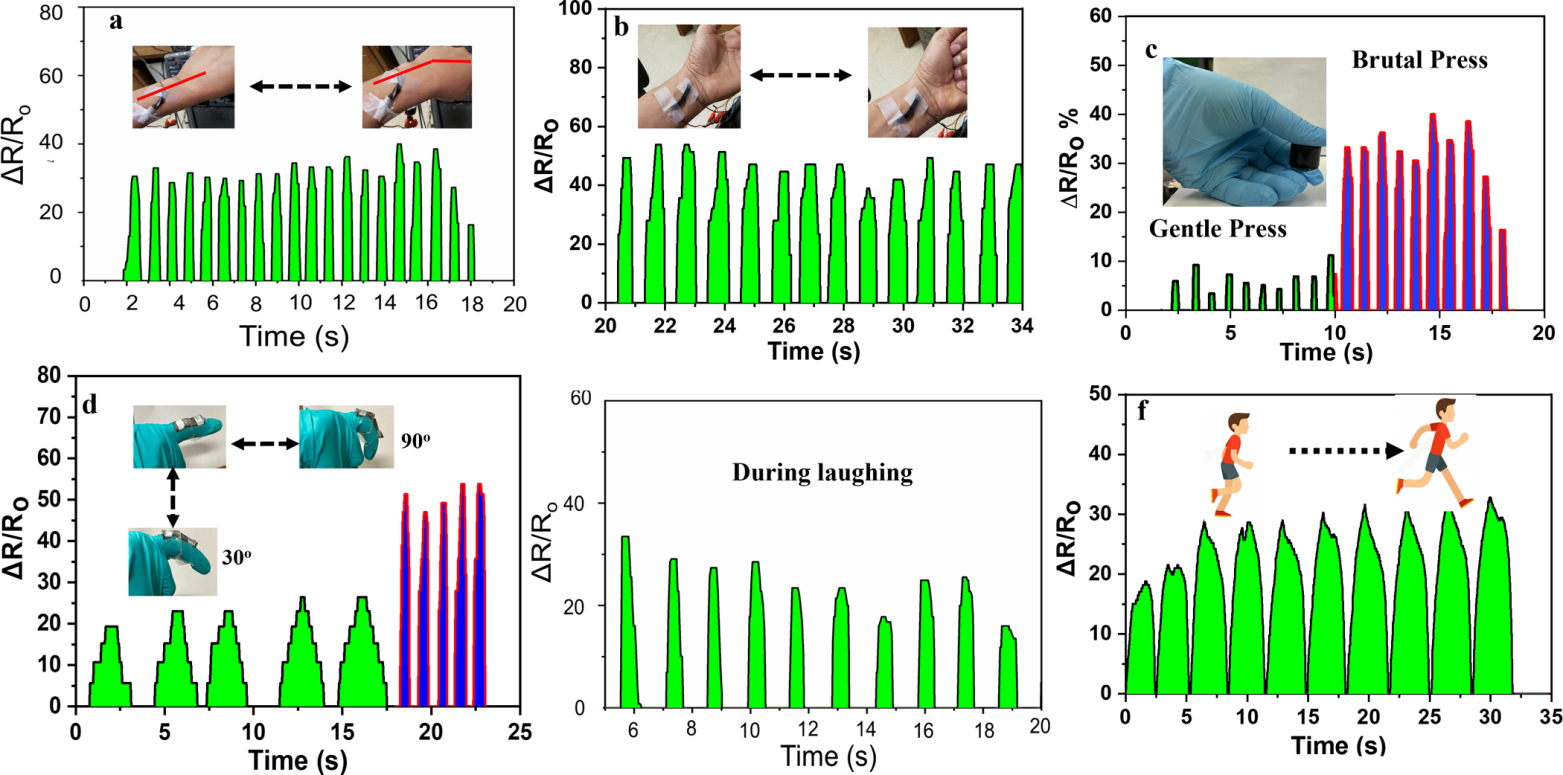
Real-time sensing performances of the PAM/DCNF4/B hydrogel served as a pressure sensor in monitoring human behavior while being adhered to the hand, elbow, and knee, respectively. Optical image of attaching a designed strain sensor on (a) wrist bending and (b) fist clenching. (c) Gentle and brutal finger compression and (d) fingers bent at different angles and (e) during laughing activity and (f) running.

The potential use of hydrogels in fields such as human–computer interaction, strain-pressure sensing, and healthcare was explored. In these investigations, the PAM/DCNF4/B hydrogel was sliced and affixed to part of human arms and face. It is shown that the PAM/DCNF4/B hydrogel has the capability of serving as a sensing substance to detect different types of body motions, such as hand gestures and facial expressions. The hydrogel’s characteristic soft and self-adhesion properties enable it to easily attach to various positions on the human body (i.e., hand and face) without the need for an adhesive. The data represented in Figure 8 demonstrates that all of the movements (i.e., running and laughing) produced consistent and distinctive signals. In Figure 8a,b,e, it can be observed that the PAM/DCNF4/B hydrogel performed well in detecting small motions, both when the hands were repositioned and when deformation occurred on the face. Moreover, the hydrogel displayed a rapid response when the fingers were bent at 0°, 30°, and 90°, which is attributed to the dynamic physical processes inside the hydrogel and its stable ion transport properties. Additionally, a pressure sensor was created to monitor finger impression events, generating consistent changes in resistance and enabling accurate discrimination between different levels of touch force (Figure 8c).

**Figure 9:**
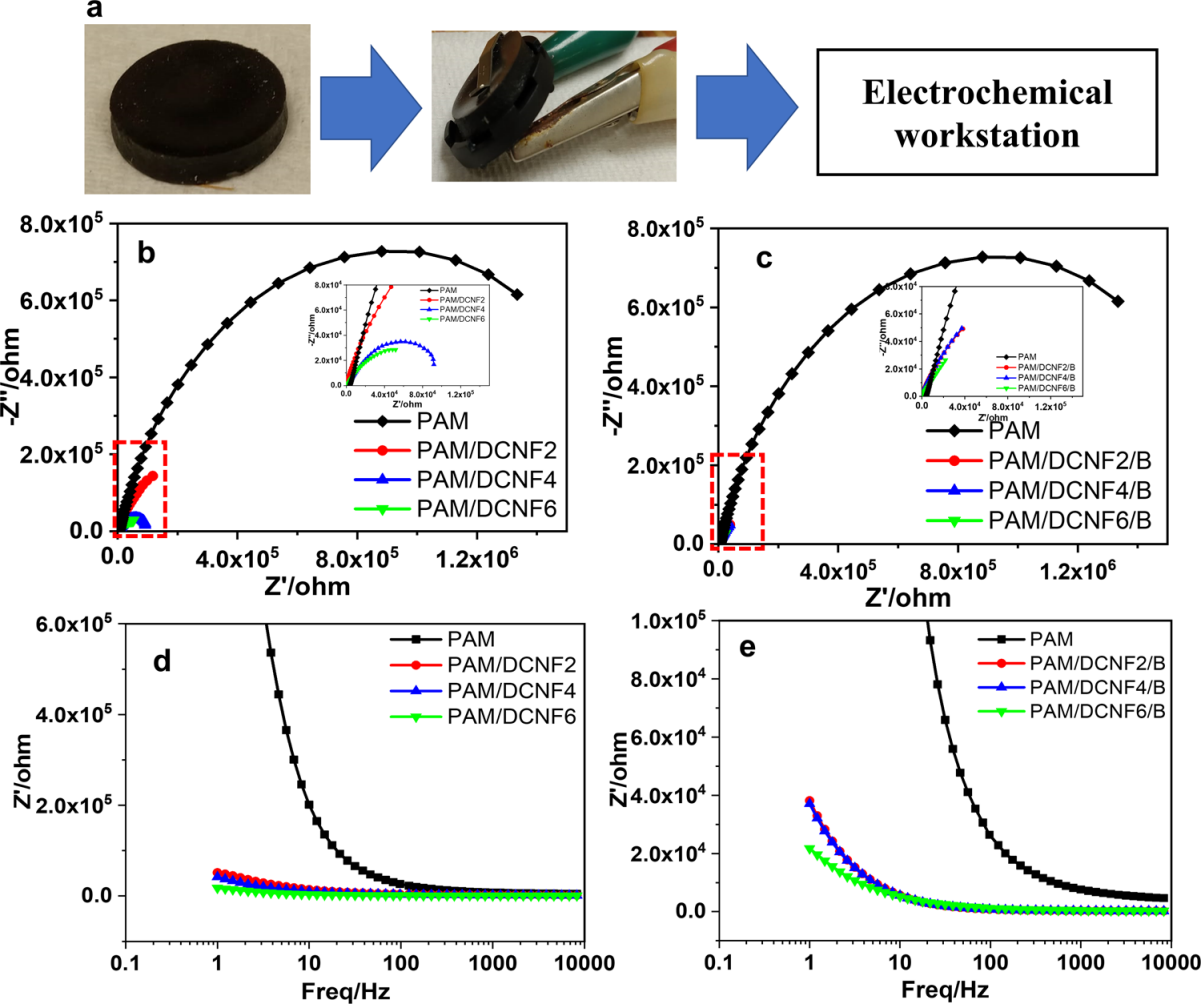
Electrochemical impedance spectra of hydrogels: (a) photograph of the sample prepared, (b, c) Nyquist plots, and (d, e) Bode plots of PAM/DCNF and PAM/DCNF/B.

The electrochemical impedance spectra (EIS) of the PAM/DCNF and PAM/DCNF/B hydrogels were measured to confirm the electrical performance of the hydrogel materials. Figure 9 represents the diameters of the prepared hydrogel sample used to study the impedance tests. The influence of DCNFs and the effect of borax on the bulk resistance are illustrated as Nyquist plots in Figure 9b,c. In particular, the DCNF content has a significant impact on the bulk resistance. The bulk resistance decreases significantly with the increase of the DCNFs in the hydrogels, which is attributed to the increment of electron migration in the hydrogels. Furthermore, PAM/DCNF/B hydrogels have a lower overall resistance compared to other PAM/DCNF hydrogels, indicating fast migration of electrons in the presence of borax. Furthermore, the EIS are presented as a Bode plot (Figure 9d,e). The impedance of all frequencies decreased when the DCNFs were increased. When DCNFs were used at the same concentrations, the PAM/DCNF/B hydrogel’s conductivity was higher than the hybrid PAM/DCNF hydrogels. The results indicate that the addition of DCNFs and borax can improve the electrical properties of PAM hydrogels, which have important applications in areas such as bioelectronics and energy storage.

### 3.7 Self-healing behavior of the hydrogel and its adhesive properties

**Figure 10:**
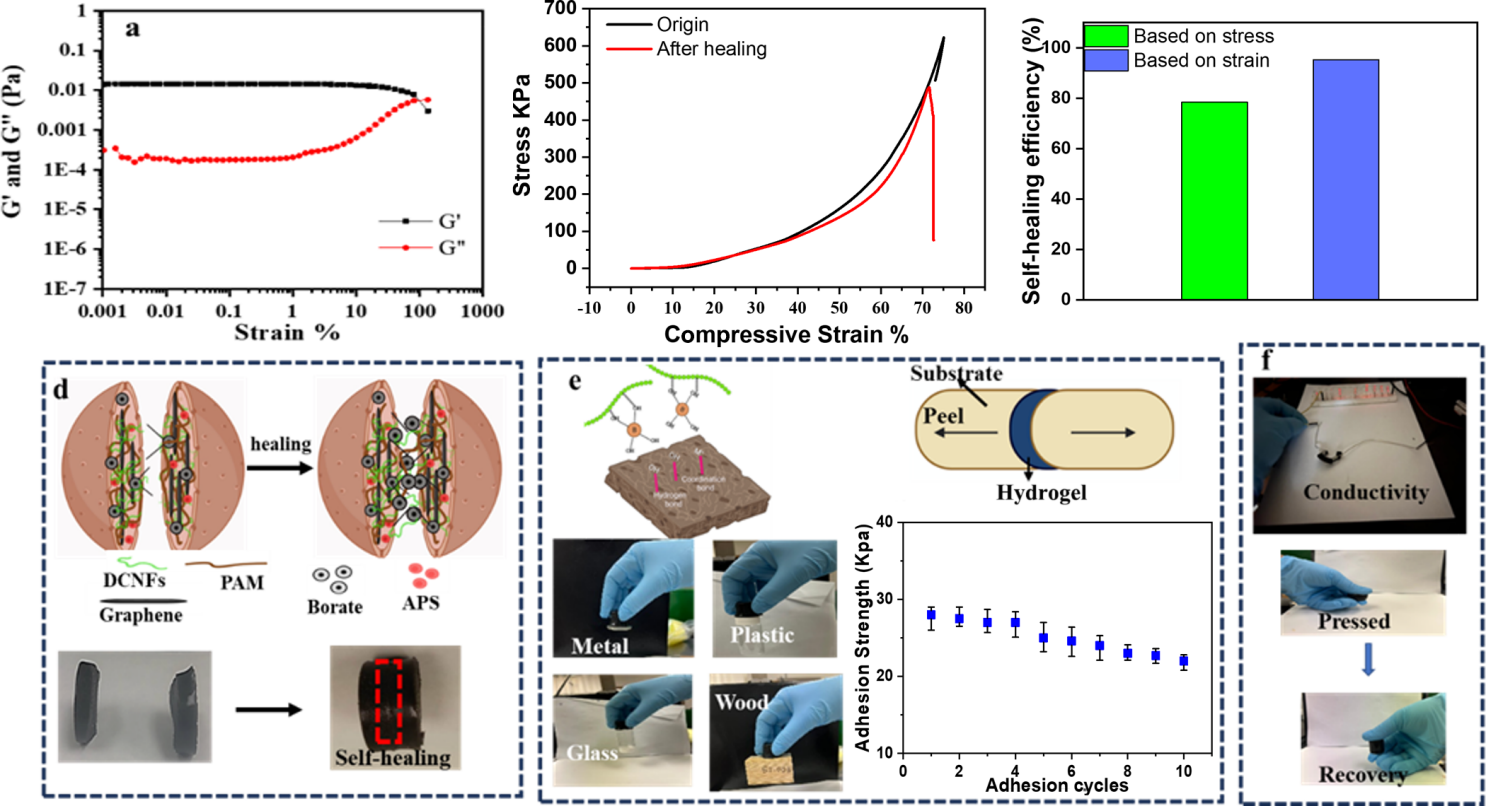
(a) Strain-dependent (ω = 10 rad/s, 25 °C) oscillatory shear rheology of the PAM/DCNF/4B hydrogel after self-healing (b) stress–strain plots of original and self-healed PAM/DCNF/4B hydrogels (c) self- healing recovery of the hydrogel base on stress and strain PAM/DCNF/4B hydrogel (d) self-heling mechanism (e) the adhesion mechanism of hydrogels to different substrates and its adhesive strength through 10 cycles (f) conductivity and recovery tests.

The strain sweep experiment was conducted to explore the self-healing characteristics of hydrogel. As displayed in Figure 10a, the gel’s moduli remained relatively stable in the linear viscoelastic region (0.001–10%) and *G*′ was consistently larger than *G*″ for strains below 100%, indicating good elasticity. However, *G*′ and *G*″ dropped dramatically when the strain exceeded the critical strain point (∼100%). In addition to its ability to restore its rheological properties, the mechanical self-healing properties, of the hydrogel were also studied. The mechanical strength of the healed hydrogel was determined through compression tests (Figure 10b,c). The stress strain curves of the healed samples were found to be similar to that of the original sample with a self-healing efficiency of 78% and 95% for stress and strain, respectively. The capability of PAM/DCNF/B hydrogels to self-healing was investigated by cutting a sample in half and then placing the fractured surfaces together. After 1 h at room temperature, the two halves healed together (Figure 10b). The self-healing behavior of these hydrogels is attributed to the reversible hydrogen bonding interactions between the borate ion and the hydroxyl groups, which permit the fractured surfaces to reconnect and form new bonds. Hydrogels have an adhesive quality due to the supramolecular interactions, such as hydrogen or coordination bonds, that form between them and various substrates. This strong bond was observed between PAM/DCNF/B hydrogels and different substrates, including wood, metal, glass, and plastic (Figure 10e). This suggests that the hydrogel has a wide range of potential applications beyond its electrical properties, such as tissue engineering, drug delivery, and wound healing. The strength of the bond between the hydrogel and the substrate is likely due to the presence of functional groups on the surface of the hydrogel that can interact with the surface of the substrate. The addition of borax to the hydrogel contributes to the adhesion properties by promoting the formation of coordination bonds with the substrate. The compressing, recovery process, and conductivity of the PAM/DCNF/B hydrogel are illustrated in Figure 10f. The results of the compression and recovery test show that the PAM/DCNF/B hydrogel has good mechanical properties and can maintain its shape even after being subjected to external forces. DCNFs and borax are known to have hydrogen bonding interactions with different hydrogen-bonding donors/acceptors on substrates, which gives for the hydrogel’s self-adhesive properties. The self-healing properties of hydrogels have been of great interest for various applications, including in the field of soft robotics, where materials can self-heal after damage are highly desirable.

## 4 Conclusions

The dual-network hydrogel developed in this work shows good mechanical, conductive, and self-healing properties, making it a suitable material for various applications, including pressure sensors and human motion monitoring. The hydrogel is formed through the combination of PAM, DCNFs, and borax with free radical polymerization of PAM hydrogels. DCNFs have a high aspect ratio and a crystalline structure, which can reinforce the PAM matrix and improve its mechanical strength. Acting as physical cross-linkers and helping form a network structure, DCNFs can also increase the cross-linking density of the hydrogel, which can further improve its mechanical properties. the DCNFs Upon 1000 loading and unloading cycles with the 50% strain rate, the compression strength decreased only about 0.8% (from 124 to 114 KPa), ensuring long-term efficiency. The maximum compressive strength reached 646 KPa, illustrating excellent mechanical properties and workability under various conditions. The role of borax primarily pertains to cross-linking the polymer chains within the hydrogel network. This cross-linking effect enhances the mechanical stability and adhesive properties of the hydrogel. However, in terms of conductivity, it is the presence of graphene that plays a crucial role. Also, the addition of borax enhanced the storage modulus (*G*′) compared with that of the hydrogel without borax, indicating a relatively stable and robust double-network of the PAM/DCNF/B hydrogels. The hydrogel-based pressure sensor demonstrated excellent pressure sensing performance (high sensitivity, high responsiveness, and wide working range) and adhesion to a variety of substrates. As a result, the developed hydrogels have a wide range of potential applications in human motion monitoring.

## Supporting Information Available

FTIR spectra of the prepared DCNFs (Figure S1); transmission electron microscopy (TEM) image and conductometric titration of the prepared DCNFs (Figure S2); thermal stability of hydrogels; TGA curves and DTG curves of hydrogels (Figure S3); effect of DCNF content of (a) PAM/DCNF and PAM/DCNF/B hydrogels on the dynamic viscoelasticity performance at 25 °C (Figure S4); effect of DCNF content of PAM/DCNF and PAM/DCNF/B hydrogels on the dynamic viscoelasticity performance at 25 °C (Figure S5); compression loading/unloading tests of cycle one for PAM/DCNF4 and PAM/DCNF4/B hydrogels at different strains (Figure S6) (PDF)
Conductivity of the PAM/DCNF4/B hydrogel (MP4)
Effect of pressure on the current of the PAM/DCNF4/B hydrogel (MP4)

## References

[ref1] Wu H.; Zhao Q.; Liang Y.; Ren L.; Ren L. Hypersensitized Strain Sensors Based on Conductive Hydrogels with Excellent Conductivity and Good Mechanical Properties. ACS Sustainable Chem. Eng. 2022, 10 (14), 4425–4437. 10.1021/acssuschemeng.1c07390.

[ref2] Kim Y.; Parada G. A.; Liu S.; Zhao X. Ferromagnetic Soft Continuum Robots. Sci. Robot. 2019, 4 (33), eaax7329 10.1126/scirobotics.aax7329. 33137788

[ref3] Liu Z.; Zhang T.; Yang M.; Gao W.; Wu S.; Wang K.; Dong F.; Dang J.; Zhou D.; Zhang J. Hydrogel Pressure Distribution Sensors Based on an Imaging Strategy and Machine Learning. ACS Appl. Electron. Mater. 2021, 3 (8), 3599–3609. 10.1021/acsaelm.1c00488.

[ref4] Wang Q.; Pan X.; Guo J.; Huang L.; Chen L.; Ma X.; Cao S.; Ni Y. Lignin and Cellulose Derivatives-Induced Hydrogel with Asymmetrical Adhesion, Strength, and Electriferous Properties for Wearable Bioelectrodes and Self-Powered Sensors. Chem. Eng. J. 2021, 414 (25), 128903 10.1016/j.cej.2021.128903.

[ref5] Caló E.; Khutoryanskiy V. V. Biomedical Applications of Hydrogels: A Review of Patents and Commercial Products. Eur. Polym. J. 2015, 65 (5), 252–267. 10.1016/j.eurpolymj.2014.11.024.

[ref6] Dutta A.; Ghosal K.; Sarkar K.; Pradhan D.; Das R. K. From Ultrastiff to Soft Materials: Exploiting Dynamic Metal–ligand Cross-Links to Access Polymer Hydrogels Combining Customized Mechanical Performance and Tailorable Functions by Controlling Hydrogel Mechanics. Chem. Eng. J. 2021, 419, 129528 10.1016/j.cej.2021.129528.

[ref7] Zhou Y.; Wan C.; Yang Y.; Yang H.; Wang S.; Dai Z.; Ji K.; Jiang H.; Chen X.; Long Y. Highly Stretchable, Elastic, and Ionic Conductive Hydrogel for Artificial Soft Electronics. Adv. Funct. Mater. 2019, 29 (1), 1806220 10.1002/adfm.201806220.

[ref8] Zhu W.; Wang J.; Sun W.; Zhou S.; He M. Preparation of Gradient Hydrogel for Pressure Sensing by Combining Freezing and Directional Diffusion Processes. Chem. Eng. J. 2023, 451 (39), 138335 10.1016/j.cej.2022.138335.

[ref9] Zhou Z.; Qian C.; Yuan W. Self-Healing, Anti-Freezing, Adhesive and Remoldable Hydrogel Sensor with Ion-Liquid Metal Dual Conductivity for Biomimetic Skin. Compos. Sci. Technol. 2021, 203, 108608 10.1016/j.compscitech.2020.108608.

[ref10] Gong Y.; Zhang Y. Z.; Fang S.; Liu C.; Niu J.; Li G.; Li F.; Li X.; Cheng T.; Lai W. Y. Artificial Intelligent Optoelectronic Skin with Anisotropic Electrical and Optical Responses for Multi-Dimensional Sensing. Appl. Phys. Rev. 2022, 9 (2), 021403 10.1063/5.0083278.

[ref11] Cheng T.; Li L.; Chen Y.; Yang S.; Yang X.; Liu Z.; Qu J.; Meng C.; Zhang Y.; Lai W. Stretchable and Self-Healing Interlocking All-in-One Supercapacitors Based on Multiple Cross-Linked Hydrogel Electrolytes. Adv. Mater. Interfaces 2022, 9 (29), 2201137 10.1002/admi.202201137.

[ref12] Cheng T.; Zhang Y.; Wang S.; Chen Y.; Gao S.; Wang F.; Lai W.; Huang W. Conductive Hydrogel-Based Electrodes and Electrolytes for Stretchable and Self-Healable Supercapacitors. Adv. Funct. Mater. 2021, 31 (24), 2101303 10.1002/adfm.202101303.

[ref13] Gong Y.; Zhang Y.-Z.; Fang S.; Sun Y.; Niu J.; Lai W.-Y. Wireless Human–Machine Interface Based on Artificial Bionic Skin with Damage Reconfiguration and Multisensing Capabilities. ACS Appl. Mater. Interfaces 2022, 14 (41), 47300–47309. 10.1021/acsami.2c14907. 36202397

[ref14] Yang J. C.; Mun J.; Kwon S. Y.; Park S.; Bao Z.; Park S. Electronic Skin: Recent Progress and Future Prospects for Skin-Attachable Devices for Health Monitoring, Robotics, and Prosthetics. Adv. Mater. 2019, 31 (48), 1904765 10.1002/adma.201904765. 31538370

[ref15] Geng L.; Liu W.; Fan B.; Wu J.; Shi S.; Huang A.; Hu J.; Peng X. Anisotropic Double-Network Hydrogels Integrated Superior Performance of Strength, Toughness and Conductivity for Flexible Multi-Functional Sensors. Chem. Eng. J. 2023, 6 (2), 142226 10.1016/j.cej.2023.142226.

[ref16] Jung H.; Kim M. K.; Lee J. Y.; Choi S. W.; Kim J. Adhesive Hydrogel Patch with Enhanced Strength and Adhesiveness to Skin for Transdermal Drug Delivery. Adv. Funct. Mater. 2020, 30 (42), 2004407 10.1002/adfm.202004407.

[ref17] Han L.; Wang M.; Li P.; Gan D.; Yan L.; Xu J.; Wang K.; Fang L.; Chan C. W.; Zhang H.; Yuan H.; Lu X. Mussel-Inspired Tissue-Adhesive Hydrogel Based on the Polydopamine–Chondroitin Sulfate Complex for Growth-Factor-Free Cartilage Regeneration. ACS Appl. Mater. Interfaces 2018, 10 (33), 28015–28026. 10.1021/acsami.8b05314. 30052419

[ref18] Zhou J.; Zhuo F.; Long X.; Liu Y.; Lu H.; Luo J.; Chen L.; Dong S.; Fu Y.; Duan H. Bio-Inspired, Super-Stretchable and Self-Adhesive Hybrid Hydrogel with SC-PDA/GO-Ca2+/PAM Framework for High Precision Wearable Sensors. Chem. Eng. J. 2022, 447 (9), 137259 10.1016/j.cej.2022.137259.

[ref19] Jing X.; Mi H.-Y.; Lin Y.-J.; Enriquez E.; Peng X.-F.; Turng L.-S. Highly Stretchable and Biocompatible Strain Sensors Based on Mussel-Inspired Super-Adhesive Self-Healing Hydrogels for Human Motion Monitoring. ACS Appl. Mater. Interfaces 2018, 10 (24), 20897–20909. 10.1021/acsami.8b06475. 29863322

[ref20] Gong J. P.; Katsuyama Y.; Kurokawa T.; Osada Y. Double-Network Hydrogels with Extremely High Mechanical Strength. Adv. Mater. 2003, 15 (14), 1155–1158. 10.1002/adma.200304907.

[ref21] Odent J.; Wallin T. J.; Pan W.; Kruemplestaedter K.; Shepherd R. F.; Giannelis E. P. Highly Elastic, Transparent, and Conductive 3D-Printed Ionic Composite Hydrogels. Adv. Funct. Mater. 2017, 27 (33), 1701807 10.1002/adfm.201701807.

[ref22] Wang H.; Li X.; Ji Y.; Xu J.; Ye Z.; Wang S.; Du X. Highly Transparent, Mechanical, and Self-Adhesive Zwitterionic Conductive Hydrogels with Polyurethane as a Cross-Linker for Wireless Strain Sensors. J. Mater. Chem. B 2022, 10 (15), 2933–2943. 10.1039/D2TB00157H. 35302157

[ref23] Zhang Y.; Liang B.; Jiang Q.; Li Y.; Feng Y.; Zhang L.; Zhao Y.; Xiong X. Flexible and Wearable Sensor Based on Graphene Nanocomposite Hydrogels. Smart Mater. Struct. 2020, 29 (7), 075027 10.1088/1361-665X/ab89ff.

[ref24] Long T.; Li Y.; Fang X.; Sun J. Salt-Mediated Polyampholyte Hydrogels with High Mechanical Strength, Excellent Self-Healing Property, and Satisfactory Electrical Conductivity. Adv. Funct. Mater. 2018, 28 (44), 1804416 10.1002/adfm.201804416.

[ref25] Holten-Andersen N.; Harrington M. J.; Birkedal H.; Lee B. P.; Messersmith P. B.; Lee K. Y. C.; Waite J. H. PH-Induced Metal-Ligand Cross-Links Inspired by Mussel Yield Self-Healing Polymer Networks with near-Covalent Elastic Moduli. Proc. Natl. Acad. Sci. U. S. A. 2011, 108 (7), 2651–2655. 10.1073/pnas.1015862108. 21278337 PMC3041094

[ref26] Zhang H.; Xia H.; Zhao Y. Poly(Vinyl Alcohol) Hydrogel Can Autonomously Self-Heal. ACS Macro Lett. 2012, 1 (11), 1233–1236. 10.1021/mz300451r. 35607147

[ref27] Tuncaboylu D. C.; Sari M.; Oppermann W.; Okay O. Tough and Self-Healing Hydrogels Formed via Hydrophobic Interactions. Macromolecules 2011, 44 (12), 4997–5005. 10.1021/ma200579v.

[ref28] Miyamae K.; Nakahata M.; Takashima Y.; Harada A. Self-Healing, Expansion-Contraction, and Shape-Memory Properties of a Preorganized Supramolecular Hydrogel through Host-Guest Interactions. Angew. Chem. Int. Ed. 2015, 54 (31), 8984–8987. 10.1002/anie.201502957. 26080301

[ref29] Wang Y.; Huang H.; Wu J.; Han L.; Yang Z.; Jiang Z.; Wang R.; Huang Z.; Xu M. Ultrafast Self-Healing, Reusable, and Conductive Polysaccharide-Based Hydrogels for Sensitive Ionic Sensors. ACS Sustainable Chem. Eng. 2020, 8 (50), 18506–18518. 10.1021/acssuschemeng.0c06258.

[ref30] He L.; Fullenkamp D. E.; Rivera J. G.; Messersmith P. B. PH Responsive Self-Healing Hydrogels Formed by Boronate–catechol Complexation. Chem. Commun. 2011, 47 (26), 7497 10.1039/c1cc11928a. PMC4526106 21629956

[ref31] Chao A.; Negulescu I.; Zhang D. Dynamic Covalent Polymer Networks Based on Degenerative Imine Bond Exchange: Tuning the Malleability and Self-Healing Properties by Solvent. Macromolecules 2016, 49 (17), 6277–6284. 10.1021/acs.macromol.6b01443.

[ref32] Yang X.; Liu G.; Peng L.; Guo J.; Tao L.; Yuan J.; Chang C.; Wei Y.; Zhang L. Highly Efficient Self-Healable and Dual Responsive Cellulose-Based Hydrogels for Controlled Release and 3D Cell Culture. Adv. Funct. Mater. 2017, 27 (40), 1703174 10.1002/adfm.201703174.

[ref33] Abouzeid R. E.; Khiari R.; Beneventi D.; Dufresne A. Biomimetic Mineralization of Three-Dimensional Printed Alginate/TEMPO-Oxidized Cellulose Nanofibril Scaffolds for Bone Tissue Engineering. Biomacromolecules 2018, 19 (11), 4442–4452. 10.1021/acs.biomac.8b01325. 30301348

[ref34] Lan X.; Ma Z.; Szojka A. R. A.; Kunze M.; Mulet-Sierra A.; Vyhlidal M. J.; Boluk Y.; Adesida A. B. TEMPO-Oxidized Cellulose Nanofiber-Alginate Hydrogel as a Bioink for Human Meniscus Tissue Engineering. Front. Bioeng. Biotechnol. 2021, 9 (5), 1–39. 10.3389/fbioe.2021.766399. PMC8602093 34805119

[ref35] Shayan M.; Gwon J.; Koo M. S.; Lee D.; Adhikari A.; Wu Q. PH-Responsive Cellulose Nanomaterial Films Containing Anthocyanins for Intelligent and Active Food Packaging. Cellulose 2022, 29 (18), 9731–9751. 10.1007/s10570-022-04855-5.

[ref36] Xu K.; Wang Y.; Zhang B.; Zhang C.; Liu T. Stretchable and Self-Healing Polyvinyl Alcohol/Cellulose Nanofiber Nanocomposite Hydrogels for Strain Sensors with High Sensitivity and Linearity. Compos. Commun. 2021, 24 (2), 100677 10.1016/j.coco.2021.100677.

[ref37] Hassan E. A.; Hassan M. L.; Oksman K. Improving Bagasse Pulp Paper Sheet Properties with Microfibrillated Cellulose Isolated from Xylanase-Treated Bagasse. Wood Fiber Sci. 2011, 43 (1), 76–82.

[ref38] Yun J.; Kim H.-I. Preparation of Poly(Vinyl Alcohol)/Poly(Acrylic Acid) Microcapsules and Microspheres and Their PH-Responsive Release Behavior. J. Ind. Eng. Chem. 2009, 15 (6), 902–906. 10.1016/j.jiec.2009.09.021.

[ref39] Abou-Zeid R. E.; Dacrory S.; Ali K. A.; Kamel S. Novel Method of Preparation of Tricarboxylic Cellulose Nanofiber for Efficient Removal of Heavy Metal Ions from Aqueous Solution. Int. J. Biol. Macromol. 2018, 119, 207–214. 10.1016/j.ijbiomac.2018.07.127. 30036619

[ref40] Gurgel L. V. A.; Júnior O. K.; Gil R. P. de F.; Gil L. F. Adsorption of Cu(II), Cd(II), and Pb(II) from Aqueous Single Metal Solutions by Cellulose and Mercerized Cellulose Chemically Modified with Succinic Anhydride. Biores. Technol. 2008, 99 (8), 3077–3083. 10.1016/j.biortech.2007.05.072. 17706418

[ref41] Li G.; Li C.; Li G.; Yu D.; Song Z.; Wang H.; Liu X.; Liu H.; Liu W. Development of Conductive Hydrogels for Fabricating Flexible Strain Sensors. Small 2022, 18 (5), 2101518 10.1002/smll.202101518. 34658130

[ref42] Zuluaga R.; Putaux J. L.; Cruz J.; Vélez J.; Mondragon I.; Gañán P. Cellulose Microfibrils from Banana Rachis: Effect of Alkaline Treatments on Structural and Morphological Features. Carbohydr. Polym. 2009, 76 (1), 51–59. 10.1016/j.carbpol.2008.09.024.

[ref43] Chen C.; Wang H.; Li S.; Fang L.; Li D. Reinforcement of Cellulose Nanofibers in Polyacrylamide Gels. Cellulose 2017, 24 (12), 5487–5493. 10.1007/s10570-017-1512-6.

[ref44] Peppas N. A.; Franson N. M. The Swelling Interface Number as a Criterion for Prediction of Diffusional Solute Release Mechanisms in Swellable Polymers. J. Polym. Sci. Polym. Phys. Ed. 1983, 21 (6), 983–997. 10.1002/pol.1983.180210614.

[ref45] Tanpichai S.; Phoothong F.; Boonmahitthisud A. Superabsorbent Cellulose-Based Hydrogels Cross-Liked with Borax. Sci. Rep. 2022, 12 (1), 8920 10.1038/s41598-022-12688-2. 35618796 PMC9134984

[ref46] Jayaramudu T.; Ko H.-U.; Kim H. C.; Kim J. W.; Kim J. Swelling Behavior of Polyacrylamide–Cellulose Nanocrystal Hydrogels: Swelling Kinetics, Temperature, and PH Effects. Materials. 2019, 12 (13), 2080 10.3390/ma12132080. 31261618 PMC6650916

[ref47] Gharekhani H.; Olad A.; Mirmohseni A.; Bybordi A. Superabsorbent Hydrogel Made of NaAlg-g-Poly(AA-Co-AAm) and Rice Husk Ash: Synthesis, Characterization, and Swelling Kinetic Studies. Carbohydr. Polym. 2017, 168 (12), 1–13. 10.1016/j.carbpol.2017.03.047. 28457428

[ref48] Jayaramudu T.; Ko H.-U.; Zhai L.; Li Y.; Kim J. Preparation and Characterization of Hydrogels from Polyvinyl Alcohol and Cellulose and Their Electroactive Behavior. Soft Mater. 2017, 15 (1), 64–72. 10.1080/1539445X.2016.1246458.

[ref49] George J.; S N S. Cellulose Nanocrystals: Synthesis, Functional Properties, and Applications. Nanotechnol. Sci. Appl. 2015, 16 (6), 45 10.2147/NSA.S64386. PMC4639556 26604715

[ref50] Dai H.; Huang H. Enhanced Swelling and Responsive Properties of Pineapple Peel Carboxymethyl Cellulose- g -Poly(Acrylic Acid- Co -Acrylamide) Superabsorbent Hydrogel by the Introduction of Carclazyte. J. Agric. Food Chem. 2017, 65 (3), 565–574. 10.1021/acs.jafc.6b04899. 28049294

[ref51] Wu G.; Panahi-Sarmad M.; Xiao X.; Ding F.; Dong K.; Hou X. Fabrication of Capacitive Pressure Sensor with Extraordinary Sensitivity and Wide Sensing Range Using PAM/BIS/GO Nanocomposite Hydrogel and Conductive Fabric. Compos. Part A Appl. Sci. Manuf. 2021, 145 (May), 106373 10.1016/j.compositesa.2021.106373.

[ref52] Li P.; She W.; Luo Y.; He D.; Chen J.; Ning N.; Yu Y.; De Beer S.; Zhang S. One-Pot, Self-Catalyzed Synthesis of Self-Adherent Hydrogels for Photo-Thermal, Antimicrobial Wound Treatment. J. Mater. Chem. B 2021, 9 (1), 159–169. 10.1039/D0TB02160A. 33226389

[ref53] Chen Z. Y.; Gao S.; Zhou R. B.; Wang R. D.; Zhou F. Dual-Crosslinked Networks of Superior Stretchability and Toughness Polyacrylamide-Carboxymethylcellulose Hydrogel for Delivery of Alendronate. Mater. Des. 2022, 217, 110627 10.1016/j.matdes.2022.110627.

[ref54] Guccini V.; Phiri J.; Trifol J.; Rissanen V.; Mousavi S. M.; Vapaavuori J.; Tammelin T.; Maloney T.; Kontturi E. Tuning the Porosity, Water Interaction, and Redispersion of Nanocellulose Hydrogels by Osmotic Dehydration. ACS Appl. Polym. Mater. 2022, 4 (1), 24–28. 10.1021/acsapm.1c01430. 35072077 PMC8765005

[ref55] Jing X.; Mi H.-Y.; Lin Y.-J.; Enriquez E.; Peng X.-F.; Turng L.-S. Highly Stretchable and Biocompatible Strain Sensors Based on Mussel-Inspired Super-Adhesive Self-Healing Hydrogels for Human Motion Monitoring. ACS Appl. Mater. Interfaces 2018, 10 (24), 20897–20909. 10.1021/acsami.8b06475. 29863322

[ref56] Cai G.; Wang J.; Qian K.; Chen J.; Li S.; Lee P. S. Extremely Stretchable Strain Sensors Based on Conductive Self-Healing Dynamic Cross-Links Hydrogels for Human-Motion Detection. Adv. Sci. 2017, 4 (2), 1600190 10.1002/advs.201600190. PMC5323873 28251045

[ref57] Huang J.; Peng S.; Gu J.; Chen G.; Gao J.; Zhang J.; Hou L.; Yang X.; Jiang X.; Guan L. Self-Powered Integrated System of a Strain Sensor and Flexible All-Solid-State Supercapacitor by Using a High Performance Ionic Organohydrogel. Mater. Horiz. 2020, 7 (8), 2085–2096. 10.1039/D0MH00100G.

[ref58] Sun P.; Li Q. Tension-Responsive Graphene Oxide Conductive Hydrogel with Robust Mechanical Properties and High Sensitivity for Human Motion Monitoring. Macromol. Mater. Eng. 2022, 7, 2200529 10.1002/mame.202200529.

