# Supplementary material for: Highly Flexible, Self-Bonding, Self-Healing, and Conductive Soft Pressure Sensors Based on Dicarboxylic Cellulose Nanofiber Hydrogels
Source: ACS Appl Polym Mater. 2023 Aug 8;5(9):7009–21. doi: 10.1021/acsapm.3c01024 (PMC10496109; doi:10.1021/acsapm.3c01024)
Supplement: Supplementary file 1 — ap3c01024_si_001.pdf [file ap3c01024_si_001.pdf]

Supporting Information

Highly Flexible, self-bonding, self-healing, and conductive soft pressure sensors based on dicarboxylic cellulose nanofiber hydrogels.

Ragab Abouzeid[a, b] Mohammad Shayan[a], Tongyao Wu [c], Jaegyoung Gwon[d], Timo A. Kärki [e], and Qinglin Wu[a*]

[a] School of Renewable Natural Resources, Louisiana State University AgCenter, Baton Rouge, LA 70803, United States

[b] Cellulose and Paper Department, National Research Centre, 33 Bohouth st., Dokki, Giza 12622, Egypt

[c] Department of Electrical and Computer Engineering, Louisiana State University, Baton Rouge, LA 70803, United States

[d] Forest Products Department, National Institute of Forest Science, 57 Hoegiro, Dongdaemun-gu, Seoul 02455, Korea

[e] Mechanical Engineering Department, Lappeenranta - Lahti University of Technology, 53850 LAPPEENRANTA, Finland

Table S1 Formulations of hydrogels containing DCNFs and GNP

Samples	AM(g)	DCNFs (g)	GNP (mg)	MBA (mg)	APS (g)	Water (mL)	Borax (Molar)
PAM	3	0.00	0.00	0.03	0.015	8.5	0
PAM/ DCNF2	3	0.06	0.03	0.03	0.015	8.5	0
PAM/ DCNF4	3	0.12	0.03	0.03	0.015	8.5	0
PAM/ DCNF6	3	0.18	0.03	0.03	0.015	8.5	0
PAM/DCNF2/B	3	0.06	0.03	0.03	0.015	8.5	0.2
PAM/DCNF4/B	3	0.12	0.03	0.03	0.015	8.5	0.2
PAM/DCNF6/B	3	0.18	0.03	0.03	0.015	8.5	0.2

Note: DCNF2, 4, and 6 represent 2, 4 and 6 % of acrylamide (AM) loading (3g), 1% graphene nanoplatelets (GNP) and N,N-methylene bisacrylamide (MBAA) loadings were controlled at 1% of AM, while ammonium persulfate (APS) loading was at 0.5% of the AM loading. A constant amount of water (8.5 ml) was used for all hydrogels. Borax loading was at 0.2 molar.

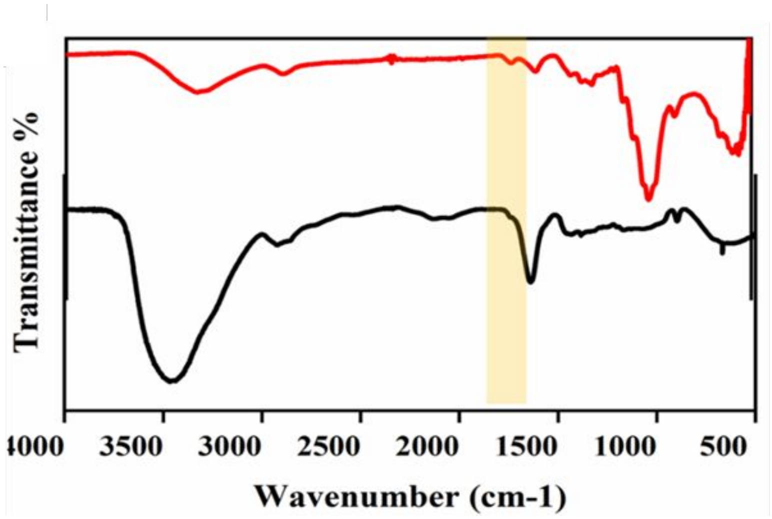

Figure S1 FTIR of cellulose pulp (black) and DCNF (red).

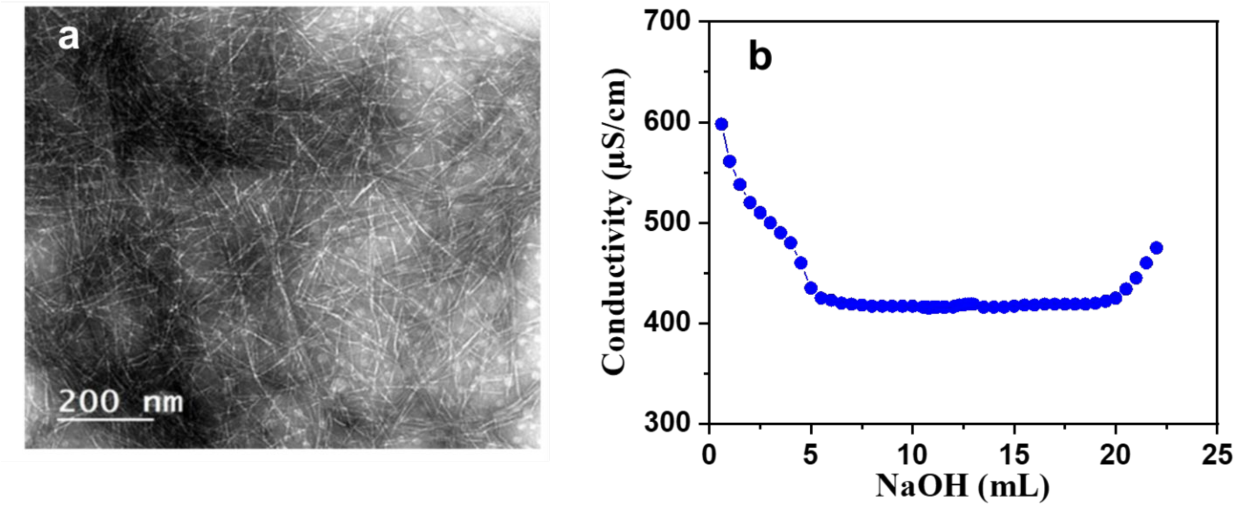

Figure S2. (a) TEM and (b) conductometric titration of the prepared DCNFs,

Thermal gravimetric analysis (TGA)

Thermal stability of PAM-based hydrogels with and without borax was studied with TGA curves shown in Figure S3 and e for all hydrogels. The residual weights of two types of hydrogels were almost the same, while the residual weight of the hydrogels increased with the content of DCNFs. It was found that the PAM, PAM/DCNF and PAM/DCNF/B hydrogels had three degradation processes. The first step had a temperature range of 25–200 °C, due to the evaporation of absorbed moisture. In the second stage from 200 °C to 350 °C, the hydrogel's covalent bonds were responsible for its structural integrity and thermal cleavage. This led to the breakdown of the hydrogel network and the release of ammonia through the imidization of PAM amide groups. Additionally, the decomposition process resulted from the cleavage and fragmentation of the cellulose backbone. In the third step over 350 °C, carbonaceous materials such as the structural backbone of the hydrogel were removed. (Figure 2d and e). Compared to PAM/DCNF, PAM was more affected at 400 °C, indicating that the polymer network formed from the combination of AM and MBA in PAM is more stable [1]. All the PAM/DCNF and PAM/DCNF/B hydrogels had similar weight losses. Figure 2f and g) shows the DTG graphs of PAM, PAM/DCNF and PAM/DCNF/B hydrogels. It can be seen that the maximum decomposition temperature of PAM was 406.0 °C, but for the PAM/DCNF2, PAM/DCNF4, and PAM/DCNF6 were 407, 409, 363°C respectively. The data values are higher than these of PAM/DCNF2/B, PAM/DCNF4/B, and PAM/DCNF6/B (372, 347, 349 °C respectively). The presence of borax played a key role in affecting early thermal degradation. In cross-linked borax hydrogels, the decrease in thermal stability may be caused by the esterification of cellulose with borax [2,3]. A lower peak height was observed for cross-linked hydrogels containing borax in DTG

curves due to thermal annealing of borax and DCNFs [2]. A hydrogel with good thermal stability can maintain its structure and properties even when being exposed to high temperatures. The thermal degradation property is important for sensor applications, as sensors may need to operate at elevated temperatures or in harsh environments.

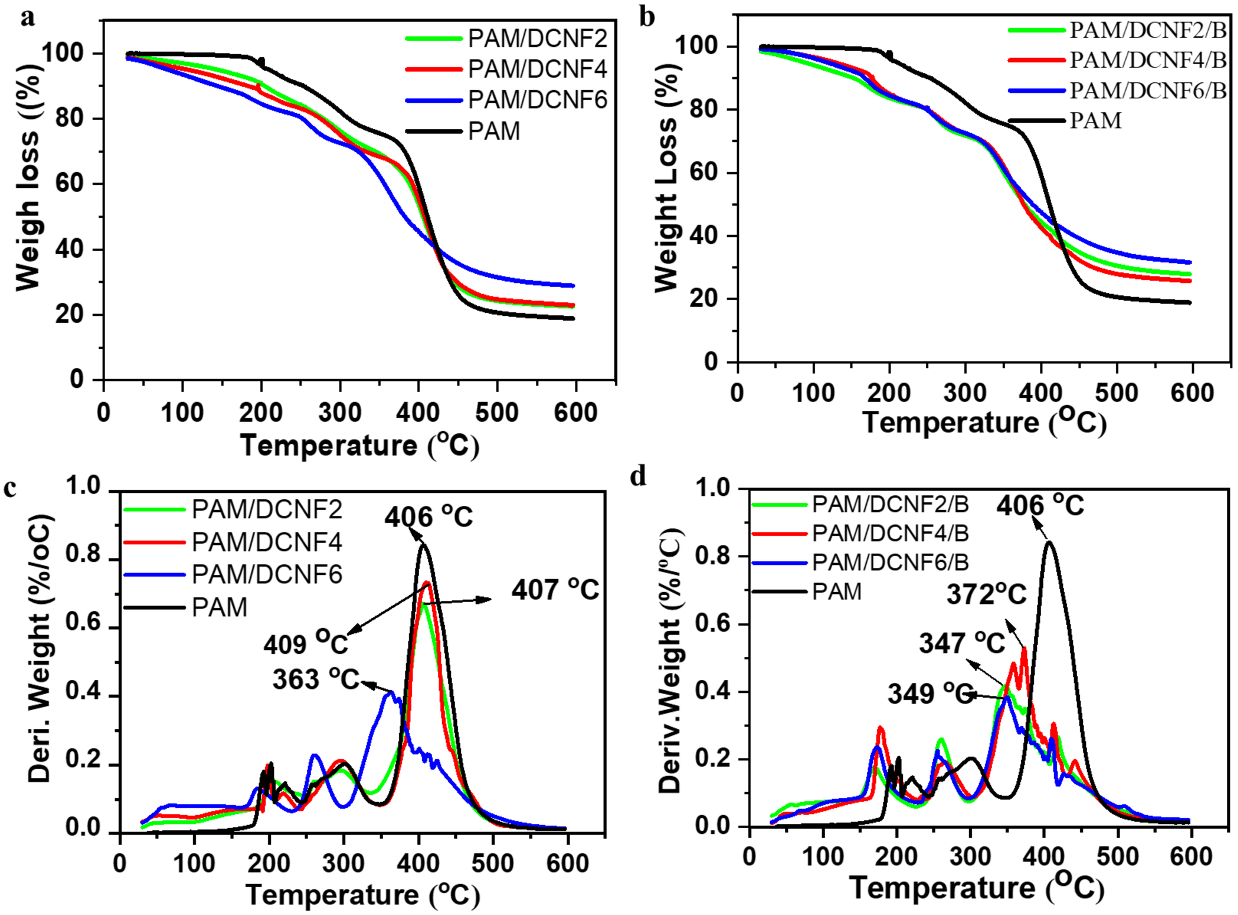

Figure S3. Thermal stability of hydrogels. TGA curves (a, b) and DTG curves (c, d) of hydrogels.

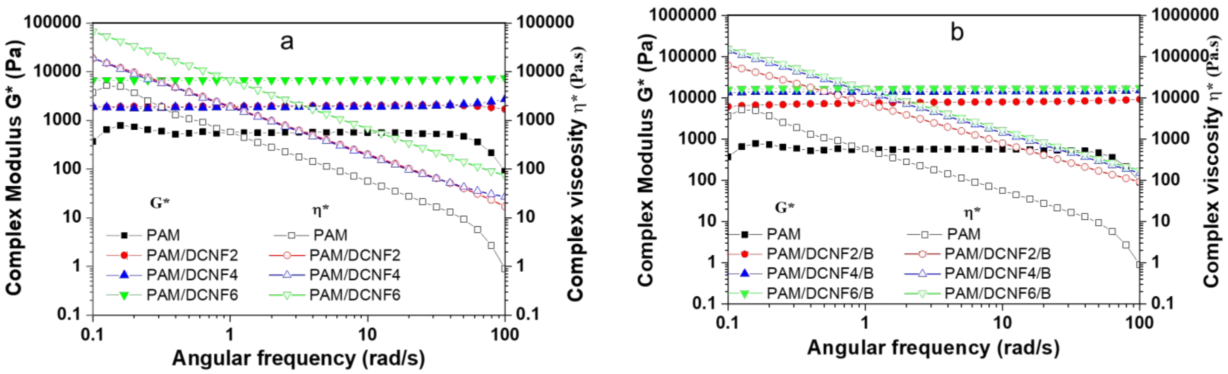

Figure S4 The effect of DCNF content (a) PAM/DCNF and PAM/DCNF/B hydrogels on the dynamic viscoelasticity performance at 25°C: Complex modulus and complex viscosity at 1.0% strain amplitude.

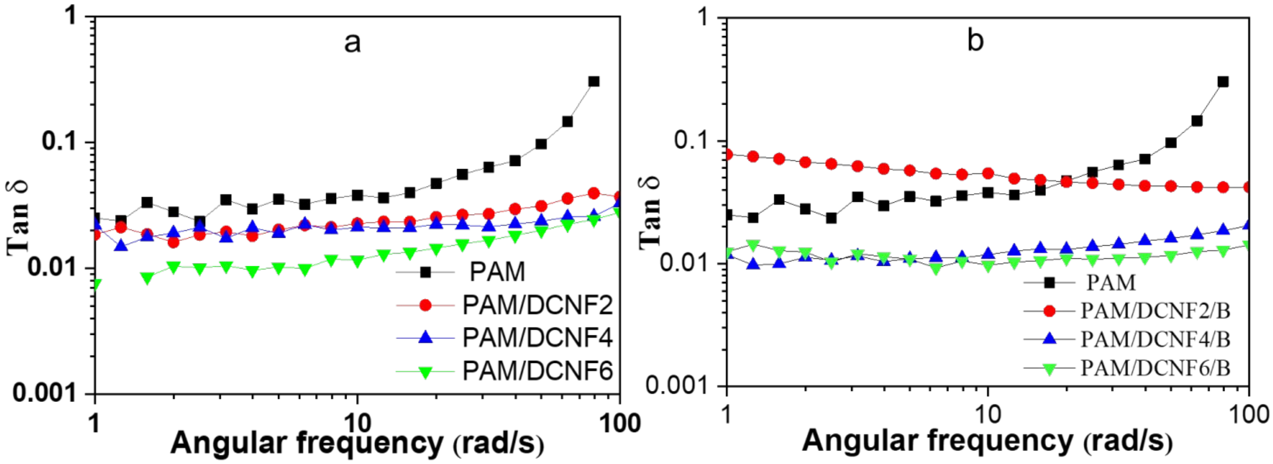

Figure S5 The effect of DCNF content of PAM/DCNF (a) and PAM/DCNF/B (b) hydrogels on the dynamic viscoelasticity performance at 25°C - Tan δ curves at 1.0% strain amplitude.

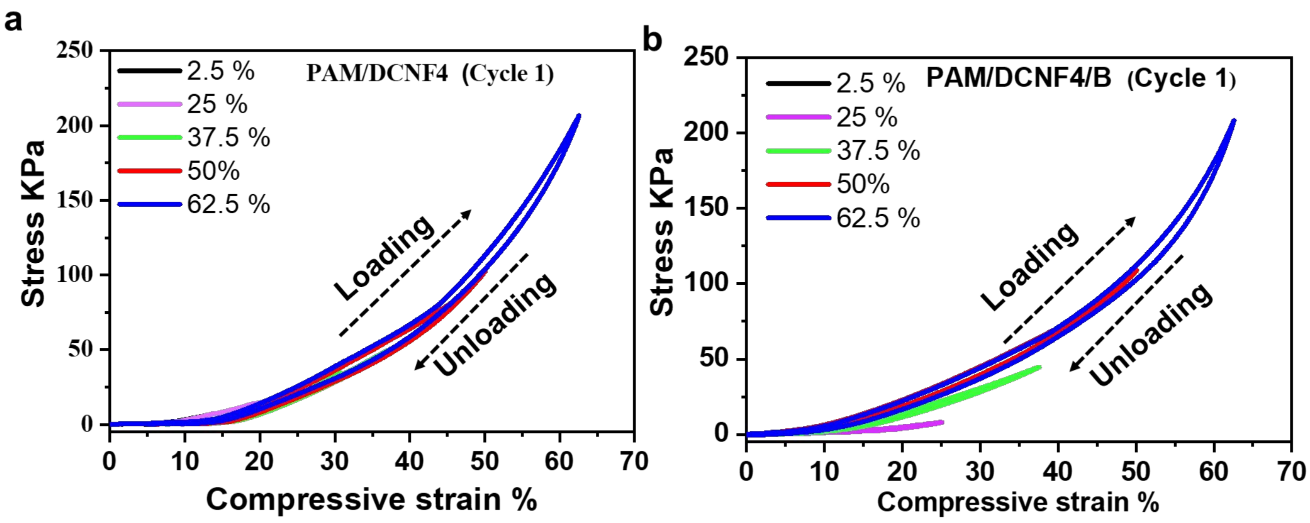

Figure S6 Compression loading–unloading tests of cycle one for PAM/DCNF4 and PAM/DCNF4/B hydrogels at different strains.

References

(1) Cheng, T.; Li, L.; Chen, Y.; Yang, S.; Yang, X.; Liu, Z.; Qu, J.; Meng, C.; Zhang, Y.; Lai, W. Stretchable and Self-Healing Interlocking All-in-One Supercapacitors Based on Multiple Cross-Linked Hydrogel Electrolytes. *Adv. Mater. Interfaces* **2022**, *9* (29), 2201137. https://doi.org/10.1002/admi.202201137.

(2) Uddin, K. M. A.; Ago, M.; Rojas, O. J. Hybrid Films of Chitosan, Cellulose Nanofibrils and Boric Acid: Flame Retardancy, Optical and Thermo-Mechanical Properties. *Carbohydr. Polym.* **2017**, *177*, 13–21. https://doi.org/10.1016/j.carbpol.2017.08.116.

(3) Tanpichai, S.; Phoothong, F.; Boonmahitthisud, A. Superabsorbent Cellulose-Based Hydrogels Cross-Liked with Borax. *Sci. Rep.* **2022**, *12* (1), 8920. https://doi.org/10.1038/s41598-022-12688-2.
